# Genome‐wide characterization and expression analysis of GATA transcription factors under combination of light wavelengths and drought stress in potato

**DOI:** 10.1002/pld3.569

**Published:** 2024-04-24

**Authors:** Emre Aksoy, Caner Yavuz, Ayten Kübra Yagiz, Necdet Mehmet Unel, Mehmet Cengiz Baloğlu

**Affiliations:** ^1^ Faculty of Arts and Sciences, Department of Biology Middle East Technical University Ankara Türkiye; ^2^ Faculty of Agricultural Sciences and Technologies, Department of Agricultural Genetic Engineering Niğde Ömer Halisdemir University Niğde Türkiye; ^3^ Plantomics Research Laboratory, Department of Genetics and Bioengineering, Faculty of Engineering and Architecture Kastamonu University Kastamonu Türkiye; ^4^ Research and Application Center Kastamonu University Kastamonu Türkiye; ^5^ Sabancı University Nanotechnology Research and Application Center (SUNUM) Sabancı University Tuzla Türkiye

**Keywords:** abiotic stress, drought, GATA, light stress, *Solanum tuberosum*

## Abstract

GATA is one of the prominent transcription factor families conserved among many organisms in eukaryotes and has different biological roles in many pathways, particularly in light regulation in plants. Although GATA transcription factors (TFs) have been identified in different crop species, their roles in abiotic stress tolerance have not been studied in potato. In this study, we identified 32 GATA TFs in potato ( *Solanum tuberosum* ) by in silico analyses, and expression levels of selected six genes were investigated in drought‐tolerant (Sante) and sensitive (Agria) cultivars under light, drought, and combined (light + drought) stress conditions. According to the phylogenetic results, StGATA TFs were divided into four main groups (I, II, III, and IV) and different sub‐groups in I and II (eight and five, respectively). *StGATA* genes were uniformly localized to each chromosome with a conserved exon/intron structure. The presence of *cis‐*elements within the *StGATA* family further supported the possible involvement in abiotic stress tolerance and light response, tissue‐specific expression, and hormonal regulation. Additional PPI investigations showed that these networks, especially for Groups I, II, and IV, play a significant role in response to light and drought stress. Six *StGATA*s were chosen from these groups for expressional profiling, and their expression in both Sante and Agria was mainly downregulated under purple and red lights, drought, and combined stress (blue + drought and purple + drought). The interactomes of selected StGATAs, *StGATA3*, *StGATA24*, and *StGATA29* were analyzed, and the accessions with GATA motifs were checked for expression. The results showed that the target proteins, cyclin‐P3‐1, SPX domain‐containing protein 1, mitochondrial calcium uniporter protein 2, mitogen‐activated protein kinase kinase kinase YODA, and splicing factor 3 B subunit 4‐like, mainly play a role in phytochrome‐mediated stomatal patterning, development, and activity. Understanding the interactions between drought stress and the light response mechanisms in potato plants is essential. It will eventually be possible to enhance potato resilience to climate change by manipulating the TFs that play a role in these pathways.

## 1 INTRODUCTION

Drought poses a significant challenge to the growth and productivity of potatoes, which are naturally adapted to temperate climates (Kikuchi et al., 2015). Insufficient water availability results in substantial losses in potato production, particularly in regions characterized by unpredictable rainfall patterns or inadequate irrigation systems (Evers et al., 2010; Thiele et al., 2010). The adverse impact of water scarcity on potato yields is projected to intensify in the coming decades, with a potential reduction of up to 32% by 2050. This decline is attributed to the progressive rise in global temperatures, escalating water requirements for agricultural activities (Hijmans, 2003). Studies focusing on improving drought tolerance in potatoes target genes with crucial roles in processes such as photosynthesis and sugar metabolism, aiming to mitigate its detrimental effects (Chen et al., 2019). The drought response mechanism in potatoes involves both ABA‐dependent and ABA‐independent pathways (Yang et al., 2019). Additionally, several transcription factor (TF) families, including MYB (Shin et al., 2011), NAC (Meng et al., 2023), DREB (Bouaziz et al., 2013), and WRKY (Moon et al., 2014), function in either a positive or negative manner within these pathways. They regulate the expression of drought‐responsive genes, further influencing the potato plant's ability to withstand drought stress. Light is critical in plant growth as it actively participates in the photosynthetic process. Plants can perceive light and utilize different groups of photoreceptors to assimilate carbon efficiently carbon (Kami et al., 2010). The photosynthetic capacity of plants is closely linked to specific wavelengths, with higher rates of photosynthesis observed in response to red light (600–700 nm), while blue light (400–500 nm) and purple‐violet light (380 nm) result in lower photosynthetic rates (Liu & van Iersel, 2021). bZIP (Filiz & Kurt, 2021), Dof (Shaw et al., 2009), MYB (Shin et al., 2011), and bHLH (Filiz & Kurt, 2021) families play crucial roles in the light response mechanism of plants. These transcription factors regulate gene expression and contribute to the plant's ability to respond and adapt to varying light conditions, thereby influencing their overall drought tolerance and growth potential.

To alleviate the detrimental effects of drought stress on plants, scientists have explored the use of different light sources with varying wavelengths and intensities to mitigate yield losses in drought‐sensitive plants. This approach aims to trigger the accumulation of antioxidative compounds under drought conditions. For instance, studies have shown that *Melissa officinalis* plants grown under red + blue and red light alone experience milder drought stress effects, attributed to abundant antioxidants and lower malondialdehyde levels, respectively (Ahmadi et al., 2020). Furthermore, exposing roquette and lettuce seedlings to red and blue light temporarily before transferring them to complete white light sources promoted stomatal activity, reducing drought's negative impact on these plants (Ginzburg & Klein, 2020). When exposed to light stress, plants exhibit similar biochemical responses, such as the production of increased reactive oxygen species (ROS), which are similar to the responses observed under drought and heat stress conditions (Szymańska et al., 2017). Moreover, these stress response mechanisms can be interconnected, and specific proteins may also provide photoprotection in potato plants under drought stress such as a recent report reviewed a likely light and ABA crosstalk during drought response in *Arabidopsis* (Mukherjee et al., 2023; Szalonek et al., 2015; Wang et al., 2023).

The GATA transcription factor family is actively involved in light response and abiotic stress mechanisms, exhibiting high conservation across eukaryotes. Identified by a characteristic type IV zinc finger domain (CX_2_CX_17‐20_CX_2_C, where C represents cysteine and X is any amino acid) and a nearby basic region, GATA factors were first identified in tobacco (Daniel‐Vedele & Caboche, 1993) and have been extensively studied in various plant species, *Arabidopsis thaliana* (Kim, Xi, & Park, 2021; Teakle & Gilmartin, 1998), rice (Reyes et al., 2004), poplar (An et al., 2014), *Noccaea caerulescens* (Milner et al., 2014), soybean (Zhang et al., 2015), apple (Chen et al., 2017), grape (Zhang et al., 2018), *Moso bamboo* (Wang et al., 2019), *Brachypodium distachyon* (Peng et al., 2021), poplar (Kim, Xi, Park, Yun, & Park, 2021), cucumber (Zhang et al., 2021), potato (Yu, Chang, et al., 2021), wheat (Du et al., 2022; Feng et al., 2022), and foxtail millet (Lai et al., 2022). The GATA transcription factor‐binding *cis*‐elements are commonly found in the promoters of genes associated with light and circadian rhythms, which are crucial for plant growth and development (Argüello‐Astorga & Herrera‐Estrella, 1998; Manfield et al., 2007). Notably, GATA2 in Arabidopsis has been identified as a positive regulator of photomorphogenesis, controlling the expression of light‐ or brassinosteroid‐responsive genes (Luo et al., 2010). The response to different light wavelengths (far‐red, red, blue, and white) has been chiefly associated with decreased expression of *GATA* genes in Arabidopsis (Manfield et al., 2007). The *GATA2* overexpressing Arabidopsis lines were phenotyped for shorter hypocotyls in the dark, far‐red, red, and blue lights (Luo et al., 2010). A member of the *B‐GATA* gene in Arabidopsis, *GNL*, is highly upregulated during 1‐h exposure to three different light sources, far‐red, red, and blue while *GATA17* expression is downregulated (Klermund et al., 2016). While our current understanding of GATA factors in abiotic stress is limited, some studies have revealed their involvement in abiotic stress responses in various plant species. For instance, subfamily I of SlGATA plays a role intomatoes' cold, drought, and salinity tolerance, and GATA1 contributes to drought tolerance in *Solanum andigenum* (Watkinson et al., 2006). A splice variant of *OsGATA23* in rice is associated with salinity and drought tolerance mechanisms (Gupta et al., 2017). In pepper, *GATA* genes display varied expression patterns in response to different abiotic stress factors, with some showing upregulation during oxidative stress but lower expression under continuous exposure to heat, drought, or salinity (Yu, Li, et al., 2021). Additionally, GATA subfamily clusters III and IV exhibit differential responses to cold, salt, and dehydration stress in *Brassica napus* (Zhu et al., 2020). In cucumber, *GATA* genes are involved in the immediate/early response to heat stress (Zhang et al., 2021).

By harnessing the potential of light treatments, researchers aim to minimize the detrimental effects of drought stress on plants. These findings highlight the cross‐regulation between drought and light stress responses, indicating shared biochemical pathways and protein functions. Although the specific roles of GATA transcription factors in potato under drought stress and light conditions have not been studied, it is known that GATA proteins play crucial functions in both mechanisms. Therefore, the objective of this study was to characterize and annotate the *GATA* genes in potato plants and determine the expression levels of six selected *GATA* genes under different light sources (white light, blue light, red light, and purple light), drought stress, and combined (light + drought) stress conditions in drought‐tolerant (Sante) and drought–sensitive (Agria) potato cultivars.

## 2 MATERIALS AND METHODS

### 2.1 Characterization of GATA proteins in potato

Putative GATA accessions in potatoes were identified using different strategies. GATA TFs from 166 different plants, including *A. thaliana*, *Oryza sativa*, *Populus trihocarpa*, *Vitis vinifera*, *Zea mays*, and *Cucumis sativus*, were collected from the Plant Transcription Factor Database (plntfdb.bio.uni‐postdam.de). Their sequences were blasted in PHYTOZOME v9.1 (www.phytozome.net) using BLASTP with default settings (Goodstein et al., 2012) and in NCBI database using TNBLASTN with default settings to find out homologous protein sequences of potato to ensure the collection of all possible potato GATA accessions from existing tools (Goodstein et al., 2012; Jin et al., 2017). All proteins encoded from the potato genome were compared with Hidden Markov Models (HMM) of conserved regions associated with the GATA motif in the Pfam (https://pfam.xfam.org) database (Finn et al., 2014). All potato GATA TFs were checked for redundant sequences and were eliminated using a redundancy tool (web.expasy.org/decrease_redundancy). Thus, the peptide sequences obtained through these steps were accepted as StGATA proteins. The conserved GATA domain (CX2CX17‐20CX2C and other GATA motif patterns) was screened in potato GATA accessions using SMART (http://www.smart.emblheidelberg.de) (Schultz et al., 1998) and Pfam (http://pfam.sanger.ac.uk) (Mistry et al., 2021). Subcellular localization was predicted with Plant‐mSubP selecting the PseAACNCCDipep prediction module using the protein queries (Sahu et al., 2020).

### 2.2 Identification of chromosomal position and exon/intron structure of StGATA TFs

StGATA proteins were positioned along 12 potato chromosomes using a BLASTP search in PHYTOZOME by executing default parameters, and these proteins were named according to their chromosomal locations. Chromosomal locations were visualized using MapChart (Voorrips, 2002).

Gene Structure Display Server (gsds.cbi.pku.edu.cn) was performed to identify exon‐intron regions of each gene (Guo et al., 2007) by matching the full‐length cDNA or CDS with the genomic region.

### 2.3 Phylogenetic tree construction and detection GATA motifs within TFs

Multiple sequence alignments were conducted using Clustal Omega with gap open (penalty: 10) and gap extensions (penalty: .1) for StGATA proteins (Larkin et al., 2007). An unrooted phylogenetic tree was created with 1,000 bootstrap replicates by the neighbor‐joining method using an alignment file (Saitou & Nei, 1987). The tree was visualized on the Interactive Tree of Life (iTOL; http://itol.embl.de/index.shtml) (Letunic & Bork, 2011). The motifs in the proteins were screened using multiple EM for motif elicitation (MEME) (http://meme.nbcr.net/meme3/meme.html) (Bailey & Elkan, 1994). Clustal Omega data were used to color the conserved amino acid sequences using Jalview 2.11.2.6 desktop version.

### 2.4 The discovery of *cis‐*regulatory elements in the promoters of *GATA*s

A region approximately 1,000 bp upstream of the transcription initiation site was selected from NCBI to discover the *cis‐*elements in the promoters of potato *GATA* genes. *Cis‐*regulatory elements (only with annotated function) were detected using PlantCARE (https://bioinformatics.psb.ugent.be/webtools/plantcare/html/) with default settings. The *cis*‐regulatory elements were visualized using TBtools (Chen et al., 2020) to cluster them by abiotic and biotic stress, light response, hormonal regulation, and tissue‐specific expression.

### 2.5 Syntheny analysis

The potato genome sequence was uploaded to the TBtool software. Then, the chromosome and location information of 32 StGATA genes were entered into the software. By one‐step MCScanX function of TBtool software interchromosmal relationships was visualized by synteny plot by using previously calculated K_A_ and Ks values.

### 2.6 Gene ontology analysis

The StGATA sequences were functionally annotated with GO classifications: biological processes, cellular components, and molecular functions using Blast2GO (http://www.blast2go.com) (Conesa & Gotz, 2008). The annotation analysis was started by BLASTp query of StGATA proteins against the non‐redundant protein database of NCBI; then, mapping and retrieval of GO terms associated with the BLAST results; ultimately, the discovery of protein queries to a previously characterized/annotated accessions. At the end of the process, the program presented three GO classifications, as mentioned above.

### 2.7 Detection of evolutionary divergence based on synonymous and non‐synonymous substitution rates

The CLUSTAL Omega multiple sequence alignment tool was used to align the GATA amino‐acid sequences and orthologous gene pairs in selected plants, such as *A. thaliana*, *O. sativa*, *Populus trichocarpa*, *V. vinifera*, and *Z. mays*, with StGATA apart from duplicate proteins encoding GATA genes. The synonymous (Ks) and non‐synonymous (Ka) substitution rates were calculated by aligning the amino‐acid sequences and their respective original cDNA sequences of GATA genes using PAL2NAL (http://www.bork.embl.de/pal2nal) (Suyama et al., 2006). T = Ks/2 λ (λ = 6.5 × 10 e‐9) formula was used to calculate the time (million years ago, MYA) to estimate the duplication and divergence of each *GATA* gene (Lynch & Conery, 2000).

### 2.8 Protein modeling of StGATA members

The protein models of discovered GATA proteins in potato were retrieved from the Protein Data Bank (https://www.rcsb.org) (Berman et al., 2000) using default settings, and these data were used to infer the 3D‐model in Phyre2 (Kelley & Sternberg, 2009) (http://www.sbg.bio.ic.ac.uk/~phyre2/html/page.cgi?id=index).

### 2.9 Protein–protein interaction and co‐expression network analysis

Protein–protein interactomes (PPIs) of each sub‐group in phylogenetic tree (Groups I to IV) were annotated in STRING DB (https://string-db.org) (Szklarczyk et al., 2015) using default settings.

The PPIs of individual *StGATA3*, *StGATA15*, *StGATA24*, *StGATA25*, *StGATA29*, and *StGATA32* were later identified using amino acid queries of these accessions in the STRING DB. The interacting protein partners of each selected *StGATA* protein were screened for *cis*‐elements approximately 1,000 bp upstream of the promoter region using the PlantCARE tool.

Co‐expression network analysis of GATA‐interactome was conducted using microarray data of *AtGATAs* from Genevestigator (Hruz et al., 2008) to show the expression profiles under light, drought, and combined stresses. The Genevestigator results were provided as heat maps.

### 2.10 In silico expression profiling of *StGATA*s under abiotic stress

The expressional data for drought (cv. Alegria, Desiree, Milva, Saturna, RNA Seq, Illumina HiSeq2000, GEO: GSM2060109), salinity (150 mM NaCI for 24 h) (cv. DM 1–3 R44, RNA‐Seq, Illumina Genome Analyzer II), mannitol (260 μM for 24 h) (cv. DM 1–3 R44, RNA‐Seq, Illumina Genome Analyzer II), and heat (35°C for 24 h) (cv. DM 1–3 R44, RNA‐Seq, Illumina Genome Analyzer II) treatments were received from Spud DB (http://spuddb.uga.edu/) by typing the keyword “GATA” into “Functional Annotation Keyword Search” tool (annotation dataset DM v6.1). The collected data were used to build a heat map using Morpheus software (https://software.broadinstitute.org/morpheus/). The rows were hierarchically clustered using default settings.

### 2.11 Plant materials, growth conditions, and treatments

Potato plantlets were obtained from the Potato Research Group at Niğde Ömer Halisdemir University. Drought‐sensitive Agria and drought‐tolerant Sante cultivars were used in the present study (Alhoshan & Ramin, 2019; Demirel et al., 2020). The potato plantlets were initially propagated with nodal culture in standard Murashige and Skoog (MS) medium supplemented with 3% sucrose and 8% phytoagar (Yagiz et al., 2020). Plants were grown under white fluorescence light (16/8 h light/dark photoperiod) and 25/16°C day/night temperatures in a growth room before stress treatment. The nodal culture was performed every 4 weeks until the desired plantlet number was reached. Nodal explant cuts from the plants at the shoot elongation stage with axillary buds were placed in MS media supplied with 20% PEG‐6000 under different light conditions, according to Verslues et al. (2006). To determine the combined effect of light wavelength and drought treatments on *StGATA* expression, four different light sources were applied to plants in transparent glass jars: white light, blue light (465 nm), red light (660 nm), and purple light (70% red and 30% blue light). The light sources were provided as LED strips with monochromatic diodes. Plants grown under white light and standard MS medium were used as the control group. Each jar included 10 potato nodes, and the experiment was conducted twice at different times with three replicates (jars) according to a randomized block design. The exposure of plants to different light sources in standard MS medium for individual light stress and plants on MS medium supplemented with 20% PEG‐6000 for individual drought stress made two other single stress groups. The stress application was maintained for 4 weeks, and shoot and root lengths were measured using calipers.

### 2.12 RT‐qPCR analysis

Total RNA was isolated from the full plants (shoot + leaves) grown under control and different stress (light and drought) treatments using an RNeasy Plant Mini Kit (QIAGEN). After removing any genomic DNA contamination by DNase I treatment (Thermo Fisher), RNAs were transcribed into cDNA using random hexamers (Thermo Fisher). The expression levels of *StGATA3*, *StGATA15*, *StGATA24*, *StGATA25*, *StGATA29*, and *StGATA32*, and their interacting accessions, M1AZB3 (NW_006239037.1) for *StGATA3*, M0ZT32 (NW_006238988.1), M0ZL05 (NW_006238985.1), and M1CSN7 (NW_006239054.1) for *StGATA24*, M1AHQ7 (NW_006238947.1) for *StGATA29* were determined using 200 ng of cDNA and LightCycler 480 SYBR Green I Master Mix (Roche) in Rotor‐Gene Q (QIAGEN). RT‐qPCR analyses were performed using three biological replicates and *Elongation Factor 1 alpha* (*StEF1α*) as housekeeping gene (Tang et al., 2017). The cycling conditions were set at 95°C for 10 min and 45 cycles for 95°C for 10 s, 56°C for 15 s, and 72°C for 20 s. The relative expression level of each gene was quantified using the 2^−ΔΔCt^ formula. Primer sequences were provided in Table S1.

### 2.13 Statistical analysis

Physiological and RT‐qPCR results were analyzed by Analysis of Variance (ANOVA) in MiniTab 19 (Pennsylvania State University, United States), followed by Tukey's post hoc test (*p* < .05).

## 3 RESULTS

### 3.1 Annotation of potato GATA TFs

GATA proteins from six different plants, *A. thaliana*, *O. sativa*, *Populus trihocarpa*, *V. vinifera*, *Z. mays*, and *C. sativus*, were compared to reveal evolutionary divergence, and their queries were searched against potato to identify and define GATA family members in *S. tuberosum*. All retrieved hits were examined for the presence of conserved GATA motifs. Potato has 32 GATA members with high (90%) sequence similarity to tomato and moderate (60%) similarity to Arabidopsis. The protein properties of each GATA TFs in potato and its homologs in tomato and Arabidopsis are listed in Table 1. The StGATA members exhibit different protein properties. Protein lengths of StGATA members ranged between 106 and 543 aa, and protein weights differ between 11.9–60.6 kDa. Instability index analysis showed that only StGATA03 and StGATA23 were stable, whereas others were considered unstable proteins. The isoelectric points of the proteins varied between 4.7 and 10.13. Phytozome identifiers of StGATA proteins are also provided in Table 2. The subcellular localization of StGATA members was predicted using Plant‐mSubP, and it was found that StGATA17 functions in Golgi, StGATA21, and StGATA24 (both from Group IV) in the cyto‐nucleus and StGATA32 in the plastid while the rest solely functions in the nucleus (Table 2). The *StGATA* genes were distributed mainly on the first chromosome, while some were also found on chromosomes 2–10.

**TABLE 1:** Details of GATA family members identified in the *Solanum tuberosum* genome.

ID	Phytozome identifier	*Solanum lycopersicum* homolog^a^			*Arabidopsis thaliana* homolog^b^			Phylogeny group	Physical position on potato genome			Protein properties				
		Phytozome identifier	Similarity (%)	E‐value	AGI number	Similarity (%)	E‐value		Chromosome	Start position (bp)	End position (bp)	Length (aa)	pI	Molecular weight (Da)	Instability index	Subcellular localization
StGATA01	PGSC0003DMP400004168	Solyc01g060490.2.1	79.3	1e‐56	AT3G06740	60.4	6.5e‐25	IIA	1	7,018,075	7,019,065	167	9.1	17,868.5	53.35	Nucleus
StGATA02	PGSC0003DMP400045152	Solyc01g090760.2.1	98.5	2.3e‐139	AT2G45050	75.9	2.2e‐67	IA	1	76,156,041	76,157,260	260	6.34	29,182.9	57.88	Nucleus
StGATA03	PGSC0003DMP400042956	Solyc01g100220.2.1	96.7	1e‐72	AT4G16141	67.8	1.1e‐25	IIA	1	83,974,894	83,976,132	151	9.94	16,382.1	33.77	Nucleus
StGATA04	PGSC0003DMP400066105	Solyc06g060940.1.1	45.9	3.9e‐13	AT4G17570	51.4	2.1e‐14	III	1	87,090,877	87,093,498	276	8.87	30,487.6	66.50	Nucleus
StGATA05	PGSC0003DMP400041157	Solyc08g007190.2.1	60.2	3.6e‐18	AT5G47140	69	7.8e‐18	III	1	87,108,608	87,117,596	328	4.7	36,090.6	62.19	Nucleus
StGATA06	PGSC0003DMP400056067	Solyc01g106030.2.1	97.3	9.6e‐173	AT3G21175	64.4	8.3e‐64	III	1	88,746,118	88,753,011	328	6.13	34,998.6	45.84	Nucleus
StGATA07	PGSC0003DMP400018893	Solyc01g106040.2.1	95.5	2.2e‐189	AT3G21175	59.2	7.5e‐47	III	1	88,760,271	88,765,700	375	4.93	40,849.4	54.41	Nucleus
StGATA08	PGSC0003DMP400005648	Solyc01g110310.2.1	97.1	3.2e‐165	AT5G66320	59.5	5.9e‐63	IF	1	92,688,321	92,689,902	309	5.92	34,283.3	51.36	Nucleus
StGATA09	PGSC0003DMP400021259	Solyc02g062380.1.1	91.8	6.1e‐123	AT3G51080	57.2	9.2e‐47	ID	1	42,803,008	42,804,209	265	5.51	29,237.6	46.14	Nucleus
StGATA10	PGSC0003DMP400027172	Solyc02g062760.2.1	90	4e‐119	AT3G50870	53.2	1.5e‐39	IIC	2	43,249,445	43,250,603	248	7.60	27394.6	57.01	Nucleus
StGATA11	PGSC0003DMP400006324	Solyc02g084590.2.1	96.9	2.6e‐67	AT5G66320	74.8	4e‐41	ID	2	61,878,669	61,880,344	127	10.13	13,994.0	65.15	Nucleus
StGATA12	PGSC0003DMP400006251	Solyc02g085190.1.1	93.7	8.4e‐126	AT3G50870	56.8	1.3e‐43	IIC	2	62,492,478	62,493,828	247	8.89	27,650.5	54.01	Nucleus
StGATA13	PGSC0003DMP400060693	Solyc03g033660.2.1	93.3	3e‐146	AT5G66320	55.7	1.5e‐50	ID	2	6,301,104	6,302,226	314	9.43	35,139.7	54.15	Nucleus
StGATA14	PGSC0003DMP400004491	Solyc03g120890.2.1	98	9.9e‐187	AT5G25830	62.3	3.3e‐69	IB	3	47,303,246	47,304,894	351	6.22	38,139.9	54.99	Nucleus
StGATA15	PGSC0003DMP400047412	Solyc04g015360.2.1	97	4.3e‐177	AT3G54810	60.7	4.9e‐59	IE	3	8,576,308	8,580,446	344	5.94	37,763.3	56.68	Nucleus
StGATA16	PGSC0003DMP400016535	Solyc04g076530.2.1	81.3	8.4e‐158	AT3G21175	60.8	3e‐52	III	4	57,138,414	57,144,212	353	5.11	38,583.9	45.48	Nucleus
StGATA17	PGSC0003DMP400047208	Solyc05g053500.2.1	93.1	3e‐98	AT3G24050	61.6	8e‐43	IC	5	57,981,482	57,983,567	254	9.60	28,905.1	46.17	Golgi
StGATA18	PGSC0003DMP400040535	Solyc05g054400.2.1	98	1.1e‐100	AT3G06740	70.7	2.4e‐31	IIA	5	59,146,530	59,148,842	197	9.74	21,550.6	62.74	Nucleus
StGATA19	PGSC0003DMP400040352	Solyc05g056120.2.1	97.9	1.6e‐172	AT3G54810	61	1.4e‐59	IE	5	60,666,558	60,671,431	328	5.87	35,876.1	56.66	Nucleus
StGATA20	PGSC0003DMP400042057	Solyc06g050170.2.1	94.5	1.4e‐84	AT3G56290	80.1	9.1e‐58	IV	6	17,472,916	17,474,051	179	9.21	20,372.9	52.16	Nucleus
StGATA21	PGSC0003DMP400046183	Solyc06g060940.1.1	96.7	3e‐281	AT4G17570.2	66.5	2.7e‐120	IV	6	40,528,193	40,536,199	538	8.54	59,898.2	55.29	Cyto‐nucleus
StGATA22	PGSC0003DMP400042076	Solyc06g075140.2.1	87.7	1.3e‐115	AT1G08010	64.2	2.1e‐39	IE	6	55,797,189	55,801,725	284	6.45	31,454.5	55.39	Nucleus
StGATA23	PGSC0003DMP400007959	Solyc07g038160.2.1	86.2	4.9e‐121	AT4G26150	50.2	4.5e‐33	IIB	7	32,077,230	32,078,760	286	9.53	31,992.9	39.70	Nucleus
StGATA24	PGSC0003DMP400035596	Solyc08g007190.2.1	98.5	3.3e‐289	AT4G17570	68	7.6e‐125	IV	8	1,025,148	1,032,685	543	7.83	60,626.9	58.82	Cyto‐nucleus
StGATA25	PGSC0003DMP400050747	Solyc08g066510.2.1	96.4	1.4e‐187	AT5G25830	63.7	1.5e‐71	IB	8	27,886,754	27,888,387	362	5.96	40,575.3	44.37	Nucleus
StGATA26	PGSC0003DMP400045557	Solyc08g077960.2.1	98.3	4.4e‐287	AT4G17570	66.7	1.6e‐122	IV	8	37,956,295	37,963,899	537	6.71	60,083.2	59.27	Nucleus
StGATA27	PGSC0003DMP400055095	Solyc09g075610.2.1	94.5	1.2e‐41	AT4G16141	74.5	1.8e‐20	IIA	9	44,969,241	44,969,972	106	9.53	11,938.1	80.33	Nucleus
StGATA28	PGSC0003DMP400020563	Solyc09g091250.2.1	99.6	2e‐149	AT3G24050.1	57.7	4.7e‐45	IC	9	49,488,945	49,491,699	278	7.60	30,654.5	63.20	Nucleus
StGATA29	PGSC0003DMP400020853	Solyc10g018560.1.1	92	5.9e‐125	AT2G45050.1	62.4	5.9e‐63	IA	10	16,117,820	16,119,292	258	7.18	29,455.5	49.07	Nucleus
StGATA30	PGSC0003DMP400014220	Solyc11g069510.1.1	95.4	1.8e‐164	AT1G08000.1	58.2	3.2e‐43	IE	11	38,752,669	38,757,856	337	9.05	37,069.5	52.83	Nucleus
StGATA31	PGSC0003DMP400008181	Solyc12g099370.1.1	98.2	1.2e‐85	AT5G49300.1	71.1	2.1e‐30	IIA	12	66,718,460	66,720,837	168	9.69	18,464.8	51.00	Nucleus
StGATA32	PGSC0003DMP400000562	Solyc12g008830.1.1	92.3	3.9e‐137	AT5G56860.1	55.1	1e‐43	IIB	12	4,393,652	4,396,134	287	9.59	31,844.6	51.81	Plastid

^a^ *Solanum lycopersicum* identifier number of the highest hit in BLASTp search in SPUD database (http://solanaceae.plantbiology.msu.edu) (Hirsch et al., 2014).

^b^ AGI number of the highest hit in BLASTp search in TAIR database (http://arabidopsis.org) (Berardini et al., 2015).

**TABLE 2:** Top 3 ontologies in gene ontology (GO) enrichment of StGATAs.

Gene groups	GO number	GO term	Fold enrichment	*P* value	GO number	GO term	Fold enrichment	*P* value
**1A**	**Biological process**				**Molecular function**			
	GO:0033468	CMP‐keto‐3‐deoxy‐D‐manno‐octulosonic acid biosynthetic process	>100	6.56E‐04	GO:0008690	3‐deoxy‐manno‐octulosonate cytidylyltransferase activity	>100	6.56E‐04
	GO:0019401	Alditol biosynthetic process	>100	1.31E‐03	GO:0000121	Glycerol‐1‐phosphatase activity	>100	6.56E‐04
	GO:0009641	Shade avoidance	>100	5.57E‐03	GO:0043136	Glycerol‐3‐phosphatase activity	>100	9.84E‐04
**1B**	GO:0010082	Regulation of root meristem growth	>100	2.74E‐03	GO:0001409	Guanine nucleotide transmembrane transporter activity	>100	1.86E‐03
	GO:0010075	Regulation of meristem growth	87.97	1.69E‐02	GO:0008446	GDP‐mannose 4,6‐dehydratase activity	>100	1.86E‐03
	GO:0048507	Meristem development	31.31	2.07E‐02	GO:0035198	miRNA binding	>100	4.33E‐03
**1C**	GO:0009859	Pollen hydration	>100	1.89E‐03	GO:0070492	Oligosaccharide binding	>100	2.37E‐03
	GO:0090549	Response to carbon starvation	>100	2.84E‐03	GO:0015098	Molybdate ion transmembrane transporter activity	>100	3.31E‐03
	GO:0048577	Negative regulation of short‐day photoperiodism, flowering	>100	3.31E‐03	GO:0030295	Protein kinase activator activity	>100	8.03E‐03
**1D**	GO:0048766	Root hair initiation	>100	3.06E‐03	GO:0042803	Protein homodimerization activity	28.92	3.43E‐02
	GO:0009926	Auxin polar transport	>100	7.75E‐05	GO:0043565	Sequence‐specific DNA binding	11.00	1.27E‐02
	GO:0009958	Positive gravitropism	>100	7.42E‐03	GO:0001067	Regulatory region nucleic acid binding	10.05	1.51E‐02
**1E**	GO:0009969	Xyloglucan biosynthetic process	>100	8.28E‐03	GO:0033843	Xyloglucan 6‐xylosyltransferase activity	>100	2.62E‐03
	GO:0009612	Response to mechanical stimulus	>100	9.15E‐03	GO:0035252	UDP‐xylosyltransferase activity	>100	4.80E‐03
	GO:0050826	Response to freezing	87.87	1.18E‐02	GO:0042285	Xylosyltransferase activity	>100	7.41E‐03
**1F**	GO:0080152	Regulation of reductive pentose‐phosphate cycle	>100	7.29E‐04	‐	‐	‐	‐
	GO:0034059	Response to anoxia	>100	1.46E‐03	‐	‐	‐	‐
	GO:0010375	Stomatal complex patterning	>100	2.00E‐03	‐	‐	‐	‐
**2A**	GO:0000028	Ribosomal small subunit assembly	95.19	1.07E‐02	GO:0016273	Arginine N‐methyltransferase activity	>100	2.91E‐03
	GO:0002181	Cytoplasmic translation	48.27	2.08E‐02	GO:0008276	Protein methyltransferase activity	48.96	2.05E‐02
	GO:0042255	Ribosome assembly	39.85	2.51E‐02	GO:0008170	N‐methyltransferase activity	41.29	2.42E‐02
**2B**	GO:0010151	Chloroplast elongation	>100	9.44E‐03	GO:0052924	All‐trans‐nonaprenyl‐diphosphate synthase (geranylgeranyl‐diphosphate specific) activity	>100	5.57E‐06
	GO:1902326	Positive regulation of chlorophyll biosynthetic process	>100	4.40E‐02	GO:0050347	Trans‐octaprenyltranstransferase activity	>100	5.57E‐06
	GO:0009416	Response to light stimulus	11.16	3.18E‐02	GO:0016987	Sigma factor activity	>100	5.35E‐03
**2C**	GO:0090057	Root radial pattern formation	>100	1.41E‐07	GO:0031176	Endo‐1,4‐beta‐xylanase activity	>100	7.63E‐03
	GO:0010226	Response to lithium ion	>100	5.45E‐03	GO:0097599	Xylanase activity	50.77	2.06E‐02
	GO:0009956	Radial pattern formation	>100	2.67E‐06	GO:0004499	N,N‐dimethylaniline monooxygenase activity	33.85	3.02E‐02
**2D**	GO:1901332	Negative regulation of lateral root development	>100	4.12E‐02	GO:1990841	Promoter‐specific chromatin binding	>100	3.28E‐03
	GO:0010102	Lateral root morphogenesis	88.30	3.87E‐04	GO:0019139	Cytokinin dehydrogenase activity	>100	5.24E‐03
	GO:0010311	Lateral root formation	84.62	1.92E‐02	GO:0070696	Transmembrane receptor protein serine/threonine kinase binding	>100	5.89E‐03
**3**	GO:0000398	mRNA splicing, via spliceosome	30.87	2.29E‐02	GO:0000829	Inositol heptakisphosphate kinase activity	>100	2.77E‐03
	GO:0006397	mRNA processing	21.54	6.78E‐04	GO:0030628	Pre‐mRNA 3′‐splice site binding	>100	8.28E‐03
	GO:0016071	mRNA metabolic process	16.88	2.77E‐03	GO:0017069	snRNA binding	75.94	1.38E‐02
**4**	GO:0032933	SREBP signaling pathway	>100	8.02E‐04	GO:0015485	Cholesterol binding	>100	8.02E‐04
	GO:0006991	Response to sterol depletion	>100	8.02E‐04	GO:0031543	Peptidyl‐proline dioxygenase activity	>100	6.40E‐03
	GO:0048317	Seed morphogenesis	>100	4.80E‐03	GO:0032934	Sterol binding	>100	7.99E‐03

### 3.2 Gene structure prediction, phylogenetic analysis/classification, and chromosomal location

**FIGURE 1:**
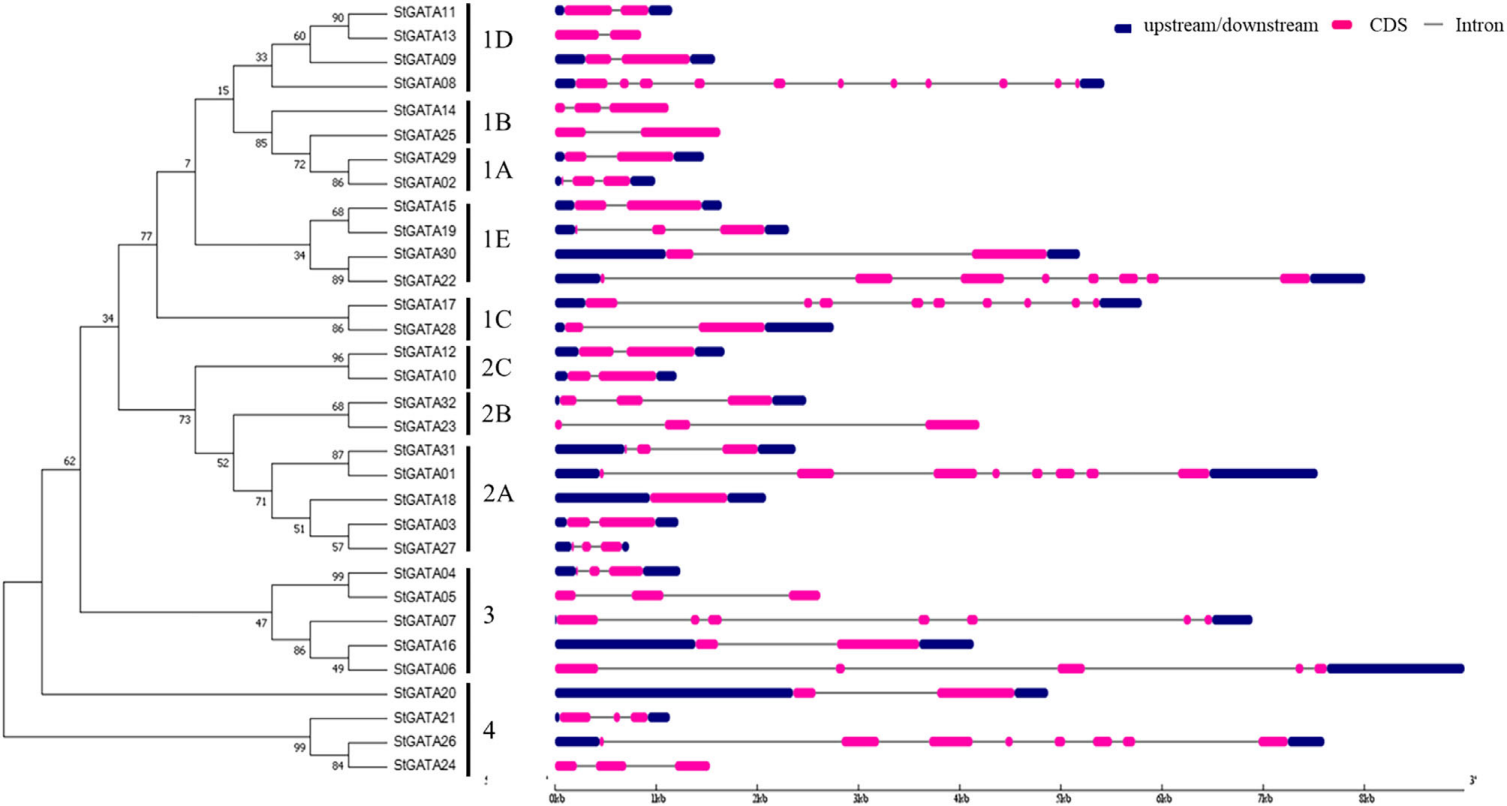
Representation of exon/intron structure for StGATA.

The exon/intron orientation for 32 *StGATA* genes was predicted using the Gene Structure Display Server. The number of exons and their positions in putative models showed substantial differences in *StGATA* genes from 1 to 10, with the maximum being 11 in *StGATA08* and the lowest 1 in *StGATA18* (Figure 1). The intron number varied between 1 and 10, the maximum was in *StGATA08*, and approximately 42% of *StGATA* genes had only one intron. *StGATA18* lacks an intron in its coding region. The categorization of TFs based on gene structure (exon/intron) did not display any distinct patterns, unlike the IA and IIC subgroups (Figure 1).

**FIGURE 2:**
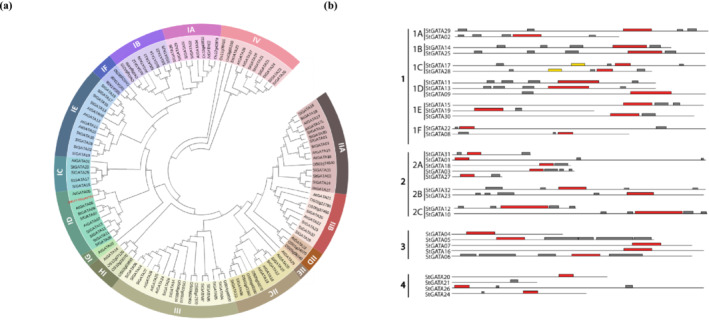
(a) Demonstration of sub‐groups among *Solanum tuberosum* , *Solanum lycopersicum* , *Oryza sativa* , and *Arabidopsis thaliana* and (b) distribution of motifs (GATA‐red, CCT motif‐yellow and others‐gray) in *S. tuberosum* .

**FIGURE 3:**
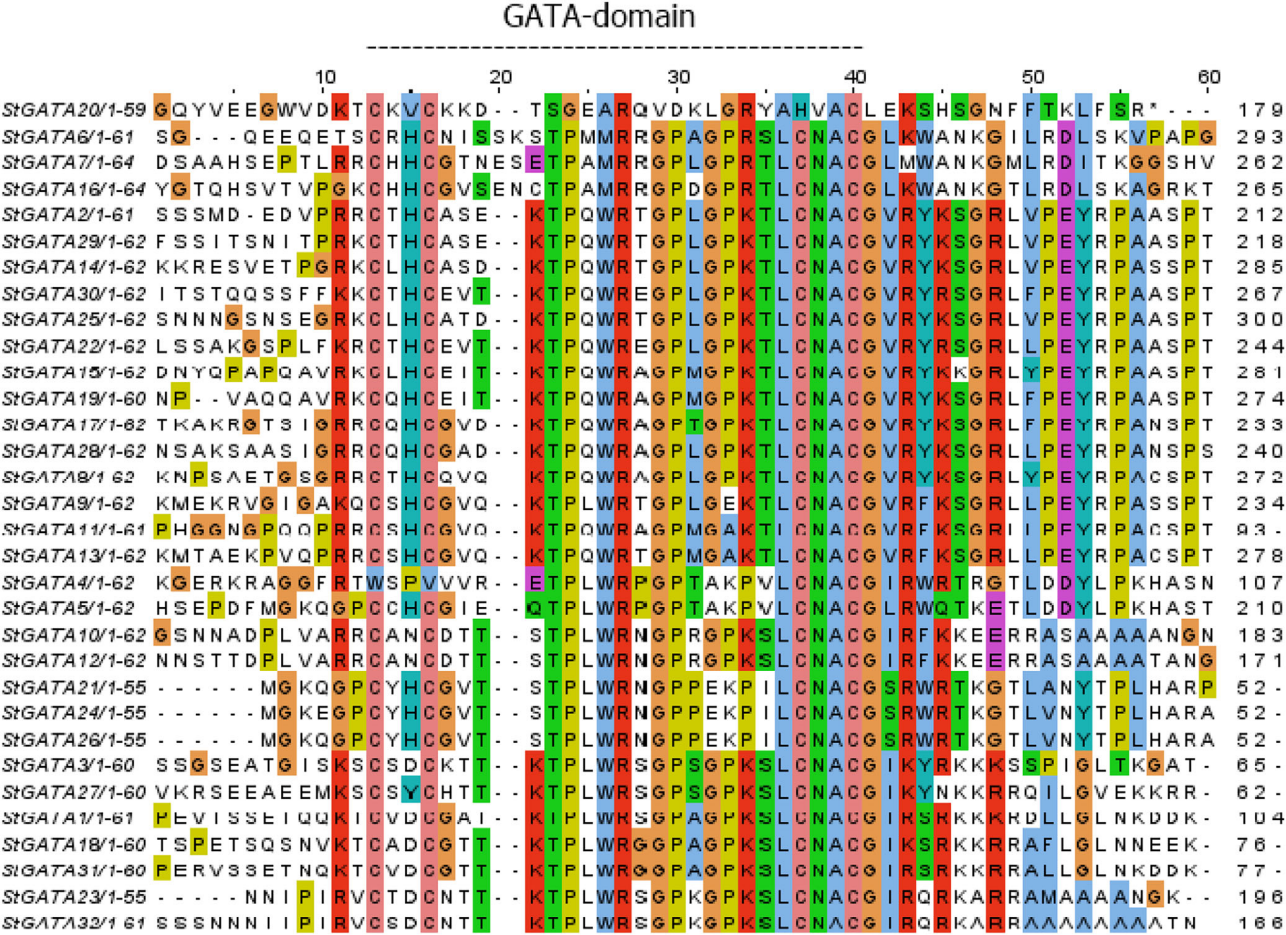
Conserved GATA domains in *Solanum tuberosum* (colored with Jalview 2.11.2.6 version).

A phylogenetic tree was constructed by aligning protein queries from *S. tuberosum*, *Solanum lycopersicum*, *O. sativa*, and *A. thaliana* to characterize and classify the subfamilies (IA, IB, IC, ID, IE, IF, IG, IH, IIA, IIB, IIC, IID, IIE, III, and IV‐4 subgroups) among these plants (Figure 2a). Members of GATA subfamilies IA, IB, ID, IIA, IIB, IIC, III, and IV clustered in all four species; however, potato lacked GATA proteins in IG and IIE subfamilies, in which only AtGATA03 and AtGATA29 evolved in *A. thaliana*. Most potato GATAs were clustered into four distinct groups: I, II, III, and IV, and subgroups, IA‐IF and IIA‐IIC (Figure 2a). Subfamily I comprised the largest group, with a total of 14 StGATA proteins, followed by nine proteins in subfamily II, five in subfamily III, and four in subfamily IV. StGATA21, StGATA26, and StGATA24 generated the outermost group in the phylogenetic tree and clustered together with SlGATA18 and SlGATA23 of tomatoes. MEME was used to annotate the conserved motifs in these StGATA sequences, and all had a single GATA motif, unlike StGATA21, which had no GATA motif. Interestingly, two StGATA proteins, StGATA17 and StGATA28, have CCT motifs and the GATA motif in their structure (Figure 2b). In addition to GATA motifs, other motifs were at different positions in all StGATA proteins (Figure 2b). In contrast to the MEME output, sequence alignment in Figure 3 showed the presence of a highly conserved GATA domain with different motif patterns among all GATA proteins, including StGATA21. All the StGATA members in Group I shared a highly conserved GATA motif, KTP(Q/L)WR‐GP‐G(P/E/A)KTLCNACGVR(Y/F)(K/R)(S/K)GRL. The motif K(T/I)PLWR‐GP‐GPKSLCNACGI(K/R)(Y/S/Q)(R/N)K(K/A)(K/R)(S/R) in Group II did not show high similarity to the other two members of this group, StGATA10 and StGATA12. Rather, these two StGATAs shared a conserved motif structure, ‐TPLWR‐GP‐(G/A/E)(P/K)(K/P)(S/V/I)LCNACG(I/L/S)(R/W)F(K/Q/R)(K/T)(E/R/K)(E/G)(R/T), with members of Groups III and IV (Figure 3).

**FIGURE 4:**
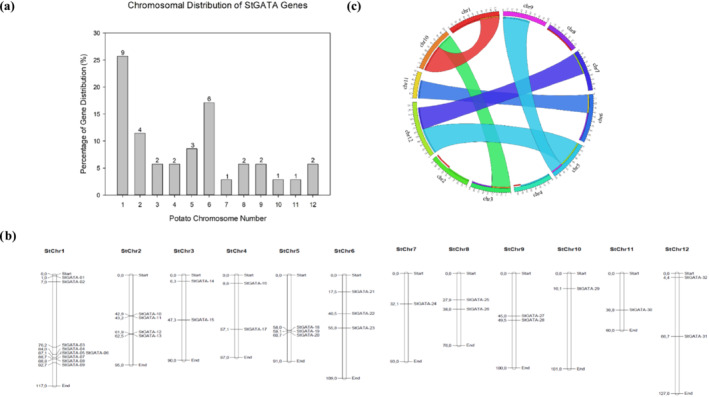
(a) Chromosomal distribution of StGATAs and (b) position of StGATAs on potato chromosomes, and (c) schematic representations of interchromosomal relationships of StGATAs. The chromosome number is indicated at the top of each chromosome.

Each potato chromosome contained at least one *GATA* gene. The highest number of genes (nine) was localized on chromosome 1, whereas the lowest was on chromosomes 7, 10, and 11 (Figure 4a). *GATA* genes were distributed to the outermost arms on chromosome 1, whereas others were uniformly condensed at the central part and on one side of the chromosome arms (Figure 4b). In the colinear segment synteny block analysis, six chromosomal pairs (chr1‐chr10, chr10‐chr3, chr9‐chr5, chr11‐chr6, chr7‐chr12, chr12‐chr5, and chr3‐chr10) were detected in potato (Figure 4c), representing potential genomic regions derived from a single ancestral genomic region.

### 3.3 Duplication gene ontology annotation and protein 3D structure analyses

**FIGURE 5:**
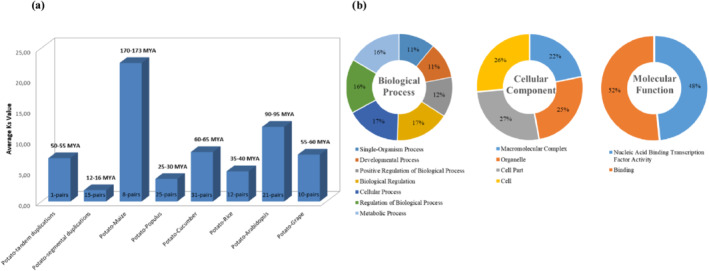
(a) Estimation of duplications and divergence of StGATAs with maize, populus, cucumber, rice, *Arabidopsis*, and grape; (b) gene ontology for StGATAs based on their biological function, cellular component, and molecular function.

Tandem (one pair) and segmental duplications (15 pairs) of GATA TFs were discovered in the potato genome. Our results show that tandem duplications diverged earlier (50–55 MYA) than segmental duplications (12–16 MYA). The evolutionary divergence of certain plants, including maize, poplar, cucumber, rice, Arabidopsis, and grapes, was also determined (Figure 5a). Accordingly, the earliest divergence was approximately 170–173 MYA for maize (8 segmental pairs) and the latest/recent was about 25–30 MYA for poplar (25 segmental pairs).

According to our gene ontology (GO) analysis, StGATA TFs have active roles in various biological processes, cellular compartments, and molecular functions (Figure 5b). They are represented the most in cellular and biological regulation and regulation of biological processes while being localized the most in organelles and cell parts with a molecular function in nucleic acid binding and transcription factor activity binding, as expected. Because the potato GATA family is divided into four major groups with slight differences in their gene structures and conserved amino acid domains, we also investigated their GO enrichment in biological processes and molecular functions (Table 2). Each subgroup was enriched in a different GO, indicating that GATA proteins were divided into subgroups according to their biological functions. Group 1 GATAs involve diverse biological processes, such as shade avoidance, root hair initiation, pollen hydration, xyloglucan biosynthesis, and response to anoxia. Group 2 GATAs are involved in ribosome assembly, chloroplast elongation, root lateral patterning, and the regulation of lateral root development. Interestingly, Group 3 GATAs are related to mRNA processing, whereas Group 4 GATAs are involved in sterol metabolism and seed morphogenesis.

The 3D protein structure was analyzed for each subgroup and representative protein 3D models are provided in Figure S1. The proteins in each subgroup showed distinct 3D structures. Groups I and II proteins had the least alpha helix (2–3) and beta‐sheet (0–3) motif numbers in their structures, respectively, whereas Group IV members had a higher number of these secondary structures, 2–7 for alpha helices and 5–6 for beta strands.

### 3.4 *cis‐*regulatory elements in the promoters of *StGATA* genes

**FIGURE 6:**
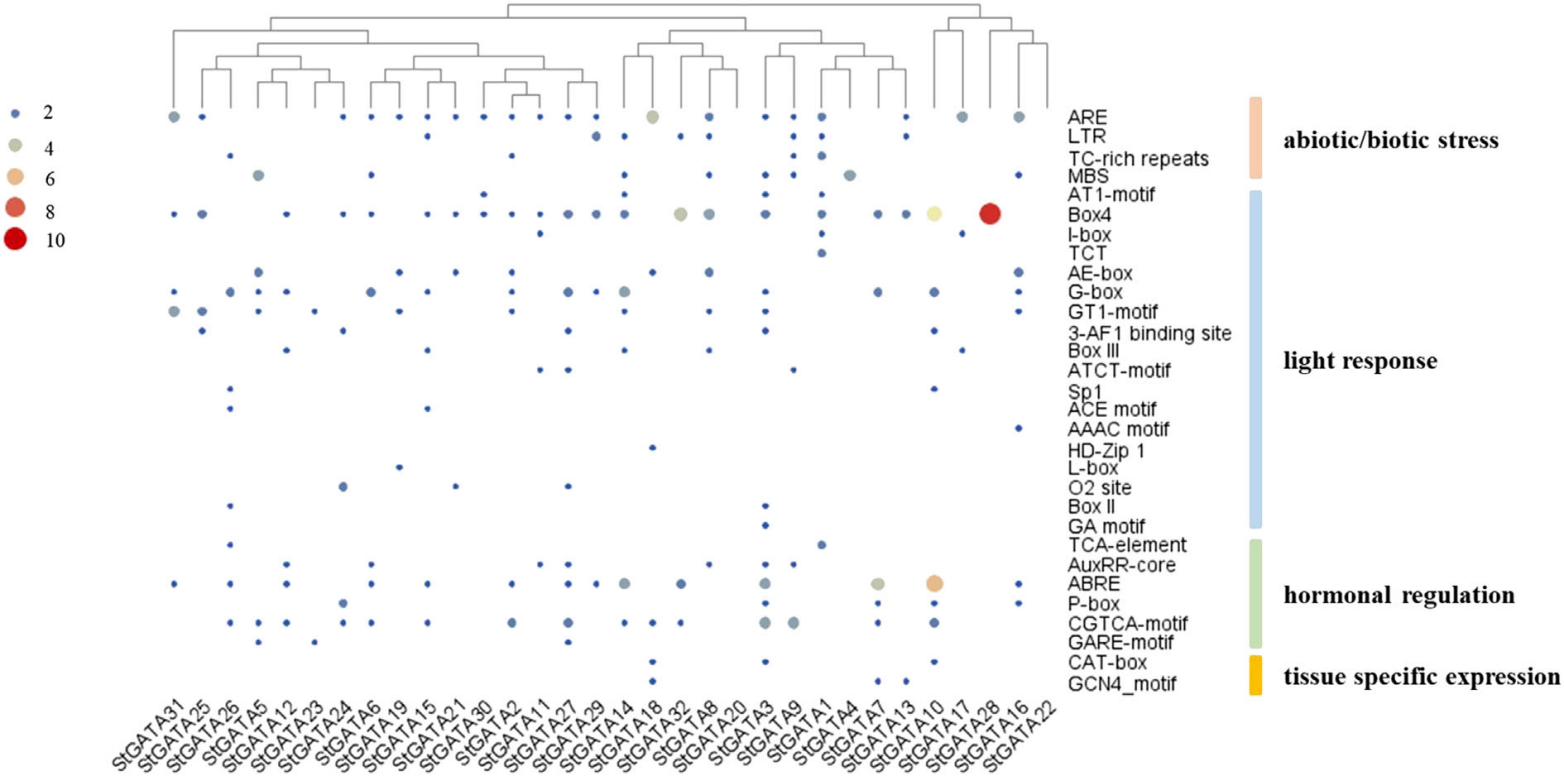
*cis* regulatory elements in the promoters of *StGATA* genes.

The discovery of *cis*‐regulatory elements in the 1,000‐bp upstream region of the promoter sequence of *StGATA* genes in PlantCARE suggests the possible regulation of GATA TFs under different conditions. Accordingly, these elements had diverse roles mostly indicating the involvement in abiotic stress tolerance (4; ARE, LTR, TC‐rich repeats, MBS) and light regulation (18; AT‐1 motif, Box 4, I‐box, TCT, AE‐box, G‐box, GT‐1 motif, 3‐AF1 binding site, Box II, GA motif, Box III, ATCT motif, Sp1, ACE motif, AAAC motif, HD‐Zip 1, L box, O2 site) (Figure 6). In addition, other *cis‐*elements functioned in tissue‐specific expression (2: CAT box, GCN4 motif) and hormonal regulation (6: TCA element, ABRE, CGTCA, AuxRR core, P‐box, GARE motif). These results suggest that *StGATA*s are regulated by many different stress‐signaling pathways.

### 3.5 Protein interaction and co‐expression network analyses of GATA‐interactome

To understand the protein interactions and co‐expression networks of StGATAs, Arabidopsis orthologs were first determined, and the protein interaction networks of each subgroup were identified individually (Figure S2). Protein–protein interaction (PPI) analysis showed that Group I had 54 interacting proteins and followed by 72, 16, and 10 interacting proteins for Groups II–IV, respectively. The PPI networks did not share any common accession numbers among the groups and subgroups. Group I PPI are functional in developmental regulation and D6PKL3 (AT3G27580) in IB and DREB26 (AT1G21910) in IE are involved in phototropism and abiotic stress tolerance (salt and drought), respectively. The GNL and GNC protein interactions of Arabidopsis with SPA4 (AT1G53090) and PIF8 (AT4G00050) in subgroup IIB play a role in photomorphogenesis, particularly in far‐red light response of the second protein.

Second, we identified the co‐expression network of Arabidopsis orthologs using the Atted II. The co‐expression network under red/blue light indicates the abundance of genes downregulated in Group II and upregulated in Group III (Figure S3). The expression of 11 *GATA* members was decreased, while the expression of three GATA genes was highly upregulated under drought and blue/red lights (Figure S4). *AtGATA2*, *AtGATA8*, *AtGATA11*, and *AtGATA12* were upregulated, and *AtGATA22* was drastically downregulated in light‐receptor mutants under the same conditions (Figure S5). Furthermore, the co‐expression network data revealed that Arabidopsis *GATA* expression was mainly decreased under drought conditions, although several GATAs, *AtGATA3*, *AtGATA15*, and *AtGATA17* showed a positive change in expression (Figure S6).

Finally, GO enrichment of these identified GATA‐interacting and/or co‐expressing genes and proteins indicated that subgroups IC and IIB might have functional roles in light response and subgroup IE might have functional roles in freezing stress (Table 2). These findings correlate well with the presence of the *cis*‐elements in *StGATA28* and *StGATA32*.

### 3.6 In silico expression profiling of *StGATAs* under abiotic stress

**FIGURE 7:**
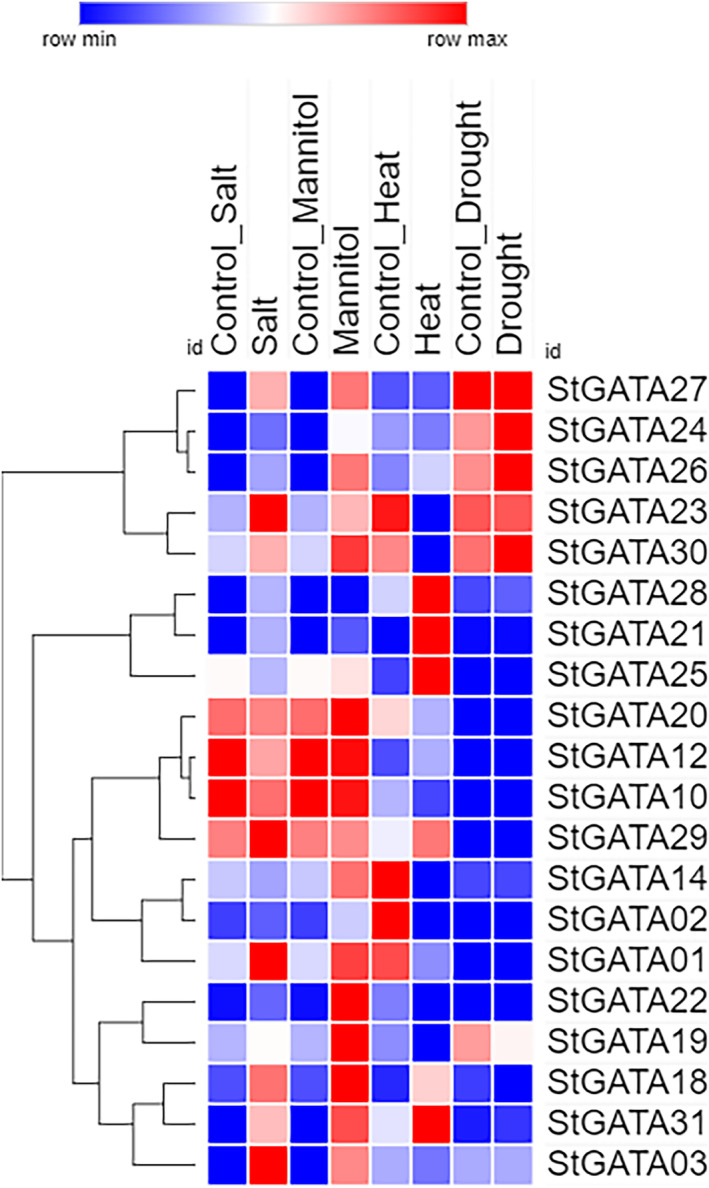
In silico expression profiling of StGATAs under abiotic stress (salt, mannitol, heat, and drought).

The in silico heat map results showed that many Group II *GATA* genes, unlike *StGATA10*, *StGATA12*, and *StGATA32*, were highly expressed in response to salt and mannitol, whereas subgroup IB members, *StGATA14* and *StGATA25* had lower expression under the same conditions (Figure 7). Several Groups I and II members and *StGATA21* showed consistent positive expression in response to heat. Interestingly, members of the same latter groups were also responsible for lower expression under heat stress (Figure 7). Potato genes under drought stress did not display a very concrete response according to the in silico heat map results, except for the higher expression of *StGATA24*, *StGATA26*, and *StGATA30* and the lower expression of *StGATA19* (Figure 7).

### 3.7 Gene expression profiling under light, drought, and combined stress of selected *StGATA*s and their interacting sequences

Two different potato cultivars, one tolerant (cv. Sante) and one susceptible (cv. Agria), was selected for the expression profiling of several selected *GATA* TFs according to in silico expression and the *cis*‐element analyses in response to different light regimes (L) (white, red, blue, and purple), drought (D), and the combined effect of these two treatments (L + D). Control and stressed plants were measured for several phenotypic traits, including shoot and root length to understand the effect of light wavelengths on drought stress responses (Figure S7). The collected data showed that Agria plants had the shortest shoot (SL) and root lengths when exposed to the combined stress, red + drought (SL: 4.7 mm), and blue + drought (SL: 6.8 mm), and no root elongation was observed for the latter treatments in Agria. Sante was relatively tolerant to light (red and purple) and particularly to drought stress respective to Agria; however, a similar response was again observed for the plants under the same combined stress as in Agria for shoot length. For root length, in vitro plantlets did not grow or develop any roots under blue + drought and purple + drought conditions. The plantlets for both cultivars were taller, especially when the light was replaced with red and purple (SL in red and purple for Agria: 53.8 and 37.8 mm, SL in red and purple for Sante: 74.2 and 77.6 mm) (Figure S7).

**FIGURE 8:**
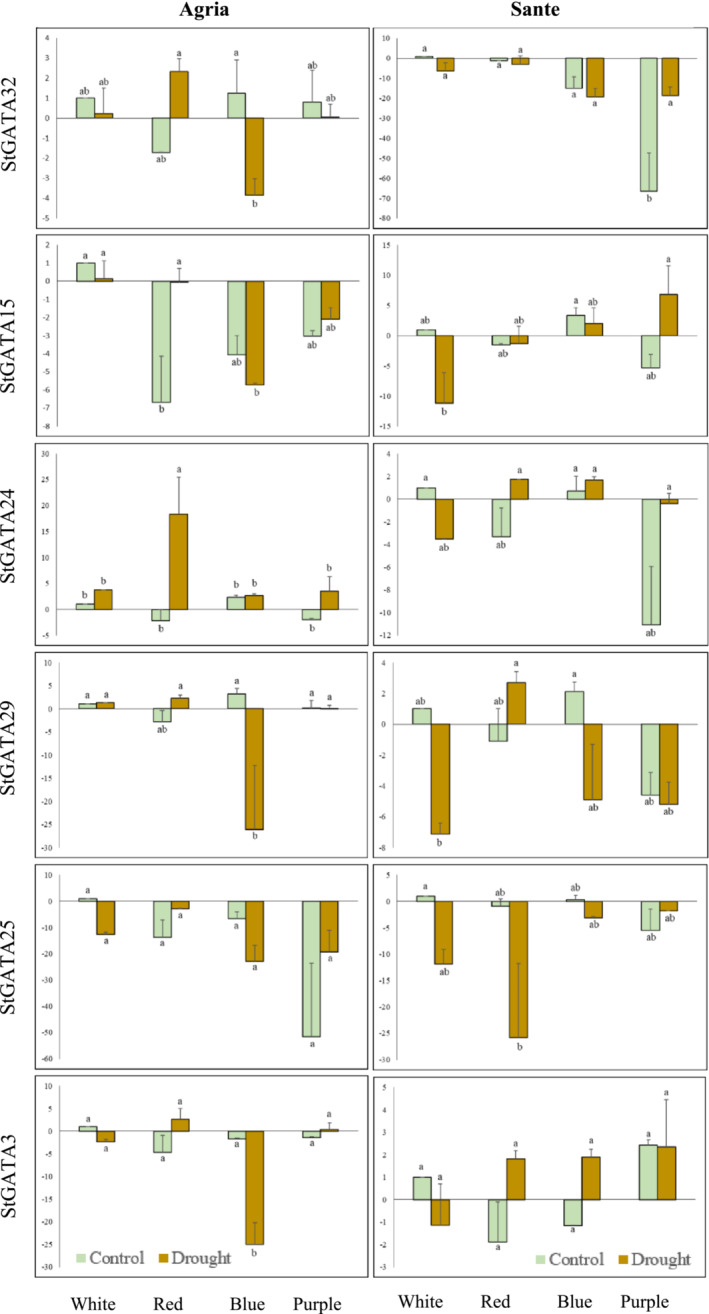
Expression of *StGATA*s in Agria and Sante under a combination of light and drought stresses.

The expression of the TFs *StGATA3*, *StGATA15*, *StGATA24*, *StGATA25*, *StGATA29*, and *StGATA32* were analyzed in potato cultivars under single and combined stresses. These candidate genes were selected regarding their (sub)‐groups (IA, IB, IE, IIA, IIB, and IV), subcellular localization (nucleus, cyto‐nucleus, and plastid), and in silico expression data. *StGATA32* was downregulated fourfold under blue + drought conditions in Agria, and a similar trend was observed in other TFs, reaching an almost 25‐fold decrease in gene expression for the same cultivar (Figure 8). In the tolerant cultivar Sante, the change towards decline for *StGATA32* was, unlikely, only significant for purple light and purple + drought. The expression considerably decreased by sixfold in red light alone for *StGATA15* in Agria; however, no significant change was observed in Sante. *StGATA24* expression slightly decreased under combined stress (red and drought stress). There was an increase in expression by almost 20‐folds in Agria, and a similar response was measured in Sante, but it was not statistically significant for the latter combined stress. In Sante, there was a dramatic change in the negative direction for the expression compared to the control in purple light alone compared to the expression value under white and without drought treatment (Figure 8). The expression of *StGATA29* in Sante significantly decreased by almost sixfold under drought conditions; however, this was not observed in Agria. *StGATA29* expression in Agria was downregulated (25‐fold) under combined blue + drought stress. On the other hand, *StGATA29* expression did not show any statistical significance under different treatments in Sante, most likely because of its tolerance. *StGATA25* expression was suppressed under all single and combined stresses in both cultivars. The mean expression difference of *StGATA25* did not reach statistical significance in cv. Sante. *StGATA3* had a very slight decrease in its expression in response to drought, and an almost 25‐fold decrease in the combined blue + drought conditions in Agria, while the opposite pattern was observed in Sante.

**FIGURE 9:**
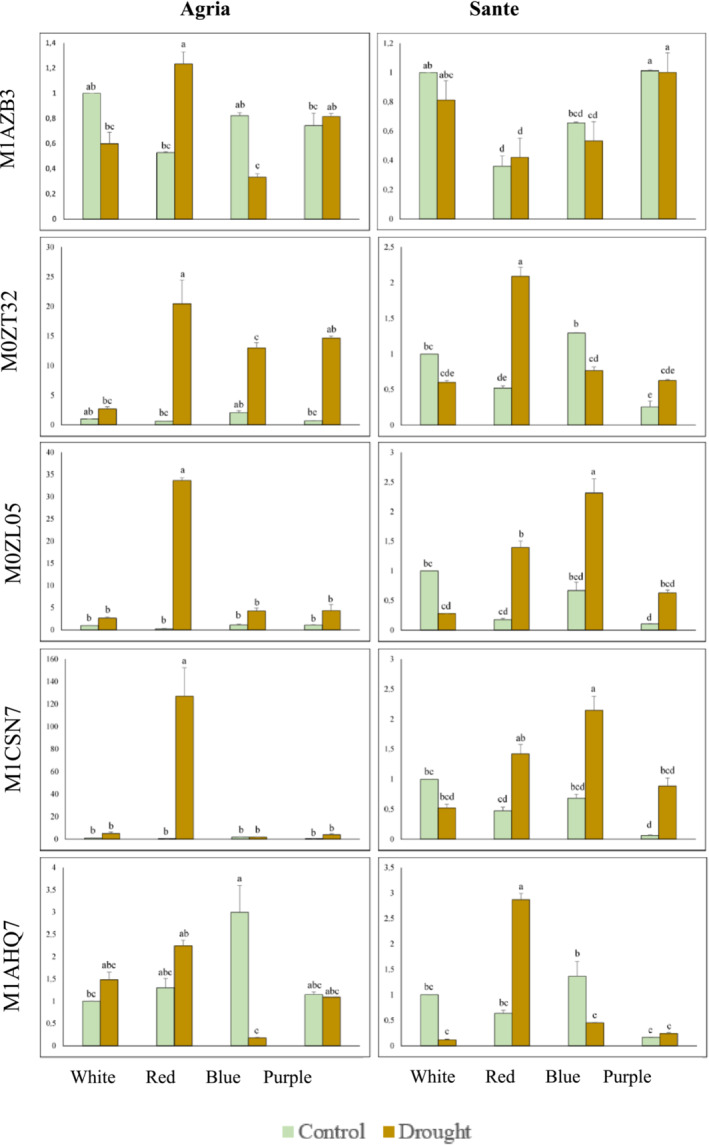
Expression of genes (and having GATA motif) interacting with *StGATA*s in Agria and Sante under individual or a combination of light and drought stresses. M1AZB3, cyclin‐P3‐1; M0ZT32, SPX domain‐containing protein 1; M0ZL05, mitochondrial calcium uniporter protein 2; M1CSN7, mitogen‐activated protein kinase kinase kinase YODA; M1AHQ7, splicing factor 3B subunit 4‐like.

Sequences interacting with the selected *StGATAs*, *StGATA3*, *StGATA15*, *StGATA24*, *StGATA25*, *StGATA29*, and *StGATA32* were identified using the STRING DB (Figure S3). No results were obtained for *StGATA32*. A total of 10 interacting proteins were found for all *GATA*s, except for *StGATA24*, for which only six were determined. We analyzed the annotations for each protein in both the STRING and NCBI databases, and their annotations are provided in Table S2. *cis*‐regulatory elements have been found in these interacting proteins, and it has been determined that these elements play a role in light and light + drought but not in drought alone. In addition, *GATA* motifs have been found in several accessions: *StGATA3* interacts with M1AZB3 (cyclin‐P3‐1), *StGATA24* interacts with M0ZT32 (SPX domain‐containing protein 1), M0ZL05 (calcium uniporter protein 2, mitochondrial), M1CSN7 (mitogen‐activated protein kinase kinase kinase YODA), and *StGATA29* interacts with M1AHQ7 (splicing factor 3 B subunit 4‐like) (Table S2). No GATA motif was found in any of the accessions interacting with StGATA15 and StGATA25. The expression levels of genes bearing the GATA motif were checked under light, drought, and light + drought conditions in both Agria and Sante (Figure 9). While the highest expression level of M1AZB3, which interacts with StGATA3, was achieved under red + drought conditions in Agria, the expression level in control conditions under white and blue light was found to be higher than that under drought conditions. No statistical difference was observed in red + drought for Sante. The expression value in control was higher for white and blue light, but no difference was observed for purple, purple + drought conditions. The expression level of M0ZT32, which interacts with StGATA24, increased under all light + drought combinations in Agria. The highest increase (20‐fold) was observed for red + drought, followed by 15‐fold induction for purple + drought and 13‐fold induction for blue + drought, and the lowest expression (threefold) was obtained for white + drought conditions in Agria (Figure 9). The highest M0ZT32 expression was in red + drought conditions for Sante. Expression levels were higher in both red + drought and purple + drought conditions compared to the control. Control expression levels were higher under white and blue light than under drought conditions (Figure 9). The expression patterns for M0ZL05 in Agria, one of the other two accessions interacting with StGATA24, were very similar to that of M0ZT32. There was a 33‐fold increase in expression level in red + drought conditions. In Sante, there was an increase in the expression level in all light + drought combinations, except white + drought. Unlike red + drought in Agria, this time the highest expression level was obtained in blue + drought conditions. The expression level increased by 127‐fold in red + drought for M1CSN7. Sante, on the other hand, gave very similar responses to that of M0ZL05. The expression level increased compared to the control under light + drought conditions, except for white light. Finally, when compared to other results, interestingly, the highest expression level was in the blue control for M1AHQ7 and StGATA29 in Agria. An increase in every light + drought treatment was observed in Agria compared to the control. The high expression level persisted in the blue control compared to the blue + drought treatment, while the highest expression was seen in the red + drought treatment for Sante (Figure 9).

## 4 DISCUSSION

The GATA transcription factor (TF) family plays diverse roles in plant growth, development, and response to abiotic stresses. Previous studies have reported the involvement of GATA TFs in drought stress in sweet potato (Zhu et al., 2022), tomato (Zhao et al., 2021), and chickpea (Niu et al., 2020), as well as their roles in light response in *V. vinifera* (Zhang et al., 2018) and poplar (An et al., 2019). Phylogenetic analyses in various plants, including Arabidopsis, soybean, apple, tomato, *Moso bamboo*, and grapes, have identified different numbers of GATA members and classified them into distinct conserved groups (Chen et al., 2017; Reyes et al., 2004; Wang et al., 2019; Zhang et al., 2015). Here, we identified 32 GATA TFs in potato grouped into four classes. While previous findings suggested variations in the number of GATA groups between dicots and monocots, our results did not align with this observation (Li, Deng, et al., 2023; Reyes et al., 2004). Several reports indicated that GATA in different plants, especially dicots, is likely to have groups between four and seven (Reyes et al., 2004). According to our results, Group I possessed the most TFs (14) compared to the other three groups (Group IV had the least TFs, 4). Based on the phylogenetic tree data, it was observed that the *StGATA* protein was not present in the IG and IIE subgroups. However, analysis revealed the presence of *AtGATA03* and *AtGATA29* in these subgroups. A literature survey showed that *AtGATA3* has been reported to functions mainly in flower and rooting formation (Zhang et al., 2013) whereas the function of *AtGATA29* remains unclear due to insufficient information; however, it is believed to be the most evolutionarily divergent of all GATA transcription factors found in Arabidopsis, as indicated by study conducted by Manfield et al. (2007). Interestingly, our classification differed from a recent study on potato by Yu, Chang, et al. (2021), indicating discrepancies in the number and grouping of GATA TFs. Moreover, two *GATA* genes identified in our study, *StGATA11* and *StGATA19*, did not show correspondence in the latter work (Yu, Chang, et al., 2021). Additionally, comparisons with another study by Saidi et al. (2021) revealed a partial overlap of GATA members in potato. While the exon intron structure of the subgroups did not display consistent gene structure patterns (especially regarding the intron number), the number of exons (2) was highly consistent and conserved compared to the variable intron number (1–10). Different gene structure patterns have been associated with the diverse functional roles of *GATA* TFs, as observed in other plant species (Feng et al., 2022; Peng et al., 2021). Understanding the tissue‐specific expression and functional roles of *GATA* TFs can provide insights into their regulatory mechanisms in potato and other plants.

We showed that the GATA members exhibited a distinct GATA motif, with two members, StGATA17 and StGATA28, belonging to the same subgroup (Group I) and featuring an additional CCT motif, as previously discussed. The presence of this CCT motif has also been observed in other GATA TFs, including Arabidopsis, and has been linked to their involvement in protein–protein interactions, suggesting a potential role in transcriptional regulation (Reyes et al., 2004; Shikata et al., 2003). Similarly, these two GATA members in potato may play a similar role. Most GATA TFs displayed a conserved CX_2_CX_20_CX_2_C domain across all subgroups, except for GATA4 and GATA20. This specific domain pattern is not commonly found among known GATA proteins, except for reports in *P. trichocarpa* (Wang et al., 2020), grapevines (Chen, Peng, et al., 2022), and Arabidopsis (Liu, 2007), and now in potato. Typically, GATA TFs possess a CX_2_CX_18_CX_2_C domain (Yu, Li, et al., 2021). Interestingly, GATA4 lacked two cysteine residues at the initial positions, while GATA20 exhibited a CX_2_CX_22_C motif pattern. To the best of our knowledge, this is the first study to report the presence of the CX_2_CX_22_C domain in any annotated GATA proteins. However, limited research exists to comprehend its potential function in plants fully.

The presence of specific *cis*‐acting elements within the promoter regions of *StGATA* TFs reinforces their involvement in abiotic stress tolerance and light regulation, aligning with findings from previous studies. Previous investigations in various plants, such as *Malus domestica* (*MdZAT17*), have identified the presence of TC‐rich repeats and other closely related *cis*‐elements associated with salinity stress (Wang et al., 2022). Additionally, the MBS *cis*‐element, found in eight out of 32 *GATA* TFs, has been implicated in the drought stress response in rice (Cheng et al., 2021). Notably, *StGATA* promoter regions possess many *cis*‐elements (18), particularly those related to light regulation, underscoring their significance in light response mechanisms. While all GATA members in potato, except *StGATA20* and *StGATA22*, contain at least one of these *cis*‐elements, *StGATA3* exhibits the highest number of *cis*‐elements (7). Consequently, *StGATA3* was selected for expression analysis to explore its response to light stress. A comprehensive understanding of various *cis*‐elements involved in light regulation has revealed the active participation of specific motifs, such as the AT1 motif in StBEL5 in potato (Chatterjee et al., 2007), the TCT motif and I‐box in SmPAL1 in *Salvia miltiorrhiza* (Zhang et al., 2020), and the AE‐box in AtPolλ in *A. thaliana* (Roy et al., 2011). These *cis*‐elements play crucial roles in mediating the light response. Moreover, the close regulatory interaction of TFs, that is, *bZIP* with G‐box (Hsieh et al., 2012), MYB with G‐, A‐, C/A‐, C/G‐, G/A‐ boxes, and ACE (Stracke et al., 2010), plant‐specific Dof with GATA box, consensus GT1 (Shu et al., 2015), GATA with ACE, L‐box, and Sp1 (Chen et al., 2017), and bHLH with Box 4 has been identified in the light response. The light signaling mechanism is primarily mediated by the interplay between the *ELONGATED HYPOCOTYL5* (*HY5*) transcription factor and *CONSTITUTIVE PHOTOMORPHOGENIC 1* (*COP1*), (functions as a ubiquitin ligase) in plants (Zhang et al., 2017). Genome‐wide identification studies have reported different TF(s) involved in recognizing the respective *cis*‐elements under light and drought stress. The *cis*‐elements reported in the drought stress response, that is, DRE (Liu et al., 2000), CATGTG (Tran et al., 2004), AATCA (Liu et al., 2022), GCC box (Zhang et al., 2010), ABRE (ACGTGG/TC) (Nakashima & Yamaguchi‐Shinozaki, 2006), and MBS (Li, Guo, et al., 2023), have been thoroughly investigated in previous studies. Furthermore, the MYB (Joshi et al., 2016), WRKY (Mare et al., 2004), and DREB (Cui et al., 2011) family of TFs has been annotated in detail for drought stress tolerance mechanisms. Strikingly, several of these *cis*‐elements, namely, GATA‐Box, have been reported to be common in light and drought responses (Li et al., 2019). Therefore, TFs that can potentially bind to these common *cis*‐elements in the promoter regions are likely to integrate the light and drought signaling pathways similarly between blue light, red light, and cold stress (Li et al., 2021). Parallel to the *cis*‐element analyses, gene ontology enrichment studies further showed that StGATA TFs have diverse roles in root development, chloroplast formation, and mRNA splicing. These members have several catalytic activities and regulation roles as primer molecular functions.

Co‐expression and PPI networks were investigated in our study for each GATA group and subgroup. As mentioned, regarding the diverse role of GATA TFs in plants, the further focus was on proteins with a particular role in response to light and drought. Group IA consisted of two proteins, AT2G42870 (PAR1) and AT5G44260 (AtTZF5), which are likely to play a role in light response and abiotic stress tolerance. PAR1, called PHYTOCHROME RAPIDLY REGULATED1, is a bHLP protein highly repressed under drought stress in Arabidopsis (Shintani et al., 2023). AtTZF5 is a zinc‐finger protein that interacts with RD21A, a cysteine protease that has a role in drought response and the immune system (Liu et al., 2022). Group IB had six interacting proteins (AT1G34110‐RGI5, AT3G27580‐ATPK7, AT4G30080‐ARF16, AT5G02260‐EXPA9, AT5G66280‐GMD1, and AT3G54770‐ARP1) reported earlier to function in either light responses, drought tolerance, or both. RGI5 is a kinase downregulated under drought stress in *Coffea arabica* (Marques et al., 2023). ATPK7 is a kinase from the D6PK family, and its active role in phototropism has been the focus of several studies investigating the crosstalk between auxin signaling and plant architecture (Willige et al., 2013). The interacting proteins, AT3G08670 (BPP6), AT4G22330 (ATCES1), AT1G09020 (ATSNF4), AT5G54830 (CYBDOMG1), AT1G22730 (MRF2) of Group IC, AT2G34650 (ABR), AT2G47260 (WRKY23) of Group 1D, and AT1G35140 (EXL1), AT5G57560 (TCH4_XTH22), AT1G21910 (DREB26), AT4G37240 (MYB), and AT2G23290 (MYB), of Group 1E have been further revealed to function actively in abiotic stress tolerance and light response. They significantly enhance tolerance against drought stress, except for AT4G37240, a member of the MYB family, which regulates gene expression under blue light in Arabidopsis (Jiao et al., 2003). Cytochromes have been previously reported to mediate the crosstalk between drought and light stress in Arabidopsis (Rao et al., 2020), and AT5G54830 in Group 1C may be responsible for similar functions in potato. Group 1G did not have any interacting proteins that were closely associated with stress or light responses. StGATA15 (Group IE), StGATA25 (Group IB), and StGATA29 (Group IA) were selected for further investigation of gene expression in response to drought, light, and combined stress conditions.

Unlike Group IIB and IIC, Group II comparably had few interacting proteins. AT3G19360 (zinc finger protein), AT5G65860 (ankyrin repeat family protein) in IIA, AT1G54330 (sugar transport), AT3G20840 (*PLT1*), AT1G12130 (*FMOGS‐OX6*) in IIC, and AT2G41510 (*CKX1*), AT2G39370 (*MAKR4*), and AT2G14960 (*GH3.1*) in IID have primary functions in regulating light response and drought tolerance. Ten proteins out of 20 that interact with the members of IIB had the same function as the latter proteins in different plant species based on previous research. They include AT1G53090 (*SPA*), AT1G17050 (*SPS2*), AT5G42760 (Leucine carboxyl methyltransferase), AT5G24120 (*SIG5*), AT3G56290 (potassium transporter), AT4G00050 (*PIF8*), AT1G66840 (*PMI2*), AT5G57180 (*CIA2*), AT3G59400 (*GUN4*), and AT2G35260 (*BCM1*). Among these proteins, the most striking protein is PIF8 (PHYTOCHROME‐INTERACTING FACTOR 3), which is highly associated with the light response and was shown to play a role in drought and salt stress tolerance (Gao et al., 2015). Therefore, we selected two genes from Groups IIA and IIB to observe the changes in expression in our treatments.

The interacting proteins of Groups III and IV were mostly involved in splicing events, biotic stress responses, development, and flowering. The only protein in III that plays a role in sugar metabolism and is likely to influence the response to drought in Arabidopsis is AT5G14270 (GTE9) (Misra et al., 2018). We did not select any genes from this group for the expression analysis. IV had two proteins, AT3G45620 (CUL4‐associated factor 8) and AT5G41410 (BEL1), respectively, which were highly upregulated under drought conditions and downregulated under light stress (Daszkowska‐Golec et al., 2018; Rossel et al., 2002). *StGATA24* from IV was chosen to observe the response under single and combined stress in potato. Taken together, our co‐expression and PPI network analyses of GATA proteins proved that GATA TFs are an essential part of the network of proteins involved in connecting the light responses and drought tolerance. Our results were further supported by a previous study where WHIRLY (WHY) was shown to interact with GATA TFs and WHY TFs are primarily involved in salt and drought response (Akbudak & Filiz, 2019). The upregulation of *ERF*, *bHLH*, *NFY*, *bZIP*, *WRKY*, and *HSF* together with *GATA* and their roles in lipid metabolism, have been recently highlighted in the cold stress response in rice and Arabidopsis (Edrisi Maryan et al. 2023), further supporting our network analyses. The crosstalk between MYB and GATA in Arabidopsis ascertained their functions in abiotic stress tolerance (Filiz & Kurt, 2021). As highlighted in previous studies, GATA TFs play significant roles in abiotic stress mechanisms across various crops. An intriguing aspect is their potential involvement in the tissue‐specific expression of genes associated with abiotic stress networks. For instance, investigations have demonstrated tissue‐specific expression of *GATA* members in potato, particularly in roots, inflorescences, and shoots (Saidi et al., 2021), while in wheat, the emphasis has been on leaf‐specific expression (Du et al., 2022). Unfortunately, conducting in silico expression analysis was challenging due to the limited information available in the solArray Potato Microarray Database. Nevertheless, the available data revealed that *StGATA20* and *StGATA32* exhibited downregulation (approximately .3‐fold change) in response to a 1‐h treatment with salicylic acid and methyl jasmonate (1 mM) in cv. Desiree. Additionally, *StGATA32* showed reduced expression (.38‐fold change) upon 1‐h application of .5 mg/ml chitin, reaching statistical significance. These expression changes under specific conditions align with previous studies that highlighted the active role of these growth regulators in various biotic and abiotic stress pathways (Cheong & Choi, 2003; Khan et al., 2015). In our study, we observed that the expression levels of six selected *GATA* members from Groups I, II, and IV were altered in light response (red and purple) (*StGATA15* and *StGATA32*, respectively) and combined stress (blue + drought) (*StGATA3* and *StGATA32*), as well as red + drought and purple + drought stress (*StGATA24*). Notably, our work is the first to demonstrate the response of *GATA* to different individual wavelengths and combined stress conditions (light + drought). While investigating GATA's involvement in different wavelengths remains limited in genome‐wide annotation studies, initial research conducted in Arabidopsis and moss has focused on several GATA members. For example, overexpression of *PpGATA1* in Arabidopsis resulted in longer hypocotyls when grown under blue light but not red light (Luan et al., 2023). Previous studies have also shown that *B‐GATA* gene expression in Arabidopsis is significantly upregulated during exposure to red, far‐red, and blue light (Klermund et al., 2016). Fortunately, there is more substantial evidence regarding the role of GATA proteins in abiotic stress tolerance mechanisms. Overexpression of *TaGATA62* and *TaGATA73* in wheat was shown to increase expression levels in response to drought and salt stresses (Du et al., 2022). Similarly, two *GATA* genes in chickpea, *CaGATA5* and *CaGATA21*, were found to be upregulated during drought (Niu et al., 2020). Numerous studies have explored the function of GATA proteins in abiotic stress tolerance mechanisms in other plants. Future studies in potato can focus on elucidating the functions of other GATA members not included in the current work, particularly concerning abiotic stress factors such as salinity and heat, as well as their combined effects. These efforts will significantly contribute to expanding our understanding of the broader roles of GATA in potato's response to abiotic stress.

To better understand the function of the selected StGATAs, the study identified StGATA interacting proteins with GATA motifs and examined their expression levels under the same conditions. Based on our findings, it has been determined that the interaction of *StGATA3* with *cyclin‐P3‐1* may be important in coordinating light and drought responses. In this study, there was an increase in the expression level under red + drought conditions, especially in the drought‐sensitive cultivar Agria; however, a similar response was not observed in the tolerant cultivar Sante. There are studies available in the literature on cyclin‐P3‐1, and its function is described by stomatal development and activity, as reported in *Leymus chinensis* (Yin et al., 2020). This could suggest the role of *cyclin‐P3‐1* and *StGATA3* in the drought response in potato, as positive transcriptional induction of each seems to stimulate the response both for sensitive and tolerant cultivars; however, the increase in expression was higher in sensitive cultivar because it experienced severe drought. Previous studies have shown that stomatal density increases in developing young leaves after exposure to drought stress (Casson & Hetherington, 2010), which could explain why the activity of *cyclin‐P3‐1* was higher in sensitive cultivar. Chlorophyll pigments are highly absorbed at red and blue lights, and the stomatal density elevates in paralel. Red light alone did not have an impact on cyclin‐P3‐1 expression; however, the combinatorial action of *StGATA3* and *cyclin‐P3‐1* could account for light and drought crosstalk. Light and drought stress function by modulating stomatal conductance in plants (Gyugos et al., 2021). *StGATA24* had three interacting proteins (*GATA* motif): SPX domain‐containing protein 1, mitochondrial calcium uniporter protein 2, and mitogen‐activated protein kinase kinase kinase YODA. All three genes showed increased transcriptional activation upon exposure to drought and red light. SPX proteins are primarily involved in biotic/abiotic stress tolerance and light responses in plants (Wang et al., 2021). *SPX* expression was reported to be higher in drought‐sensitive sesame cultivars in an earlier study (Baghery et al., 2022). Similar results to those of the present study were obtained for *SPX* in potato. In literature, there is only one report that has shown the synchronous activity of *cyclin‐P3‐1* and *SPX*, yet in response to aluminum accumulation (Fan et al., 2019). It is suggested that *SPX* might indirectly affect stomatal activity via phosphorus mechanism (Khan et al., 2023). The other interacting protein of *StGATA24*, mitochondrial calcium uniporter protein 2, is a transport protein that mediates the Ca^+2^ ion balance in chloroplasts (Teardo et al., 2019). This protein is likely to play a regulatory role in the Ca^+2^ dependent ABA signaling pathway during drought response (Pirasteh‐Anosheh et al., 2016). The highest increase was estimated in the red + drought treatment for Agria, similar to the other two interacting proteins. The association of GATA and mitochondrial calcium uniporter protein 2 with light and drought stress has not been reported before, yet a recent RNA‐seq study in pea embryos under Ca^+2^ deficiency revealed that GATA expression was downregulated under deficient conditions (Chen, Yang, et al., 2022). There is a cross‐talk between Ca^+2^ and phosphate mechanism as Ca^+2^ aggregates with phosphate and generates an insoluble compound (Stael et al., 2012). This might suggest an interplay between SPX and mitochondrial calcium uniporter protein 2; however, the role of SPX in light and drought responses should be further investigated. *StGATA24* also interacts with the mitogen‐activated protein kinase kinase kinase YODA. *YODA* has been found to be a negative regulator of stomatal development in *Populus* (Hamanishi et al., 2012) and Arabidopsis (Kang et al., 2009). Tripathi et al. (2019) described a pathway where *B‐GATA* and *YODA* coordinate stomatal development and patterning through phytochromes (phyA/phyB). Phytochromes suppress the activities of COP1 (constitutive photomorphogenesis protein 1) and PIF4 (phytochrome‐interacting factors) upon red and white light exposure. Another study reported that stomatal aperture increased in phyB‐overexpressing plants under red and blue light (Wang et al., 2010). The last protein, splicing factor 3 B subunit 4‐like, which interacts with *StGATA29*, had no information regarding its function in the literature. However, in our study, the expression was found to be highly upregulated in red + drought conditions in both Agria and Sante.

Overall, understanding the interplay between drought stress and light response mechanisms in potato is essential for developing strategies to enhance their drought tolerance. Researchers aim to improve the potato plant's ability to withstand and recover from drought‐induced stress by targeting drought‐stress‐responsive genes involved in photosynthesis, sugar metabolism, and light response pathways. The functional characterization of several StGATAs particularly *StGATA3*, *StGATA24*, and *StGATA29* is prone to further research for the elucidation of the crosstalk between light and drought stress in potato. Identifying and manipulating key transcription factors within these pathways hold promise for enhancing potato resilience in the face of changing climatic conditions, contributing to sustainable potato production and food security.

## AUTHOR CONTRIBUTIONS

Emre Aksoy and Mehmet Cengiz Baloğlu conceived and designed the research. Emre Aksoy, Caner Yavuz, Ayten Kübra Yagiz, Necdet Mehmet Unel, and Mehmet Cengiz Baloğlu conducted the in silico analyses. Emre Aksoy and Caner Yavuz conducted the gene expression experiments. Emre Aksoy, Caner Yavuz, Ayten Kübra Yagiz, Necdet Mehmet Unel, and Mehmet Cengiz Baloğlu analyzed the data. Emre Aksoy and Caner Yavuz wrote the manuscript. Emre Aksoy and Mehmet Cengiz Baloğlu edited the manuscript. All authors read and approved the manuscript.

## CONFLICT OF INTEREST STATEMENT

The authors declare that they have no conflict of interest.

## Supporting information


**Data S1.** Peer Review.

**Table S1.** Primer information about selected GATA members and the interacting sequences in potato for qRT‐PCR.
**Table S2.** Annotation of selected *StGATA* interacting proteins.
**Figure S1.** Protein models for *StGATA* proteins from different sub‐groups in potato.
**Figure S2.** Protein–protein interaction analysis of *GATAs* by sub‐groups.
**Figure S3.** Protein–protein interaction analysis of selected *StGATAs*, a) *StGATA3*, b) *StGATA15*, c) *StGATA24*, d) *StGATA25*, and e) *StGATA29*.
**Figure S4.** Hierarchical clustering of co‐expression network genes under high red/blue light. Expression patterns of all the genes in different groups of co‐expression network drawn by genes interacting with *AtGATA*s were determined in the microarray experiment on high red/blue light for different durations (GEO series of GSE31587). Red boxes indicate the genes with the most significant high or low expression that were used for further analyses.
**Figure S5.** In silico expression pattern of Arabidopsis *GATA*s under high blue‐ and red‐light intensities as well as light‐receptor mutants. a) Heat map of AtGATAs under high blue/red‐light intensities. b) Heat map of AtGATAs in light‐receptor mutants.
**Figure S6.** In silico expression pattern of Arabidopsis *GATA*s under drought. Heat map of *AtGATA*s under differnet drought experiements were drawn by Genevestigator. Hierarchical clustering was completed by Manhattan distance with optimal leaf‐ordering of genes and conditions.
**Figure S7.** Physiological responses of two potato cultivars to a combination of drought and light. a) Shoot length (mm). b) Root length (mm) (C: control, D: drought, W: white, R: red, B: blue, P: purple).

## Data Availability

The data is available from the corresponding author upon reasonable request.
