# Supplementary material for: Genome‐wide characterization and expression analysis of GATA transcription factors under combination of light wavelengths and drought stress in potato
Source: Plant Direct. 2024 Apr 24;8(4):e569. doi: 10.1002/pld3.569 (PMC11042883; doi:10.1002/pld3.569)
Supplement: Supplementary file 2 — Table S1. Primer information about selected GATA members and the interacting sequences in potato for qRT‐PCR. Table S2. Annotation of selected StGATA interacting proteins. Figure S1. Protein models for StGATA proteins from different sub‐groups in potato. Figure S2. Protein–protein interaction analysis of GATAs by sub‐groups. Figure S3. Protein–protein interaction analysis of selected StGATAs, a) StGATA3, b) StGATA15, c) StGATA24, d) StGATA25, and e) StGATA29. Figure S4. Hierarchical clustering of co‐expression network genes under high red/blue light. Expression patterns of all the genes in different groups of co‐expression network drawn by genes interacting with AtGATAs were determined in the microarray experiment on high red/blue light for different durations (GEO series of GSE31587). Red boxes indicate the genes with the most significant high or low expression that were used for further analyses. Figure S5. In silico expression pattern of Arabidopsis GATAs under high blue‐ and red‐light intensities as well as light‐receptor mutants. a) Heat map of AtGATAs under high blue/red‐light intensities. b) Heat map of AtGATAs in light‐receptor mutants. Figure S6. In silico expression pattern of Arabidopsis GATAs under drought. Heat map of AtGATAs under differnet drought experiements were drawn by Genevestigator. Hierarchical clustering was completed by Manhattan distance with optimal leaf‐ordering of genes and conditions. Figure S7. Physiological responses of two potato cultivars to a combination of drought and light. a) Shoot length (mm). b) Root length (mm) (C: control, D: drought, W: white, R: red, B: blue, P: purple). [file PLD3-8-e569-s002.docx]

| **Accession ID** | **Name** | **Primer** | **Primer Sequence** | **Amplicon Size** | **Start Position** | **Length** |
| --- | --- | --- | --- | --- | --- | --- |
| PGSC0003DMP400000562 | StGATA-32 | **Left Primer** | GCTCGCCTTGCTTTTCTTT | 150 bp | 71 bp | 19 bp |
|  |  | **Right Primer** | CAGCAACAGCTCTTCCAACA |  | 220 bp | 20 bp |
| PGSC0003DMP400004491 | StGATA-15 | **Left Primer** | GTTTCTGGTGGAGATGGACAA | 175 bp | 59 bp | 21 bp |
|  |  | **Right Primer** | TGAACTGCAGGTTTTGAACG |  | 233 bp | 20 bp |
| PGSC0003DMP400007959 | StGATA-24 | **Left Primer** | CTCGTCTTGCCTTCCTTTGT | 172 bp | 60 bp | 20 bp |
|  |  | **Right Primer** | TTGGGAGATCAAAAGCAACA |  | 231 bp | 20 bp |
| PGSC0003DMP400020853 | StGATA-29 | **Left Primer** | GGAGTTTGAAGGGAAATTATTGG | 178 bp | 7 bp | 23 bp |
|  |  | **Right Primer** | TTACCAACCACCTCCTACCG |  | 177 bp | 20 bp |
| PGSC0003DMP400050747 | StGATA-25 | **Left Primer** | ATTACTCGGCTACCGGGATT | 156 bp | 66 bp | 20 bp |
|  |  | **Right Primer** | CTCATTGCTTGCACCATCTG |  | 221 bp | 20 bp |
| PGSC0003DMP400045152 | StGATA-03 | **Left Primer** | CGACGCAGTATTGGAACTGA | 182 bp | 10 bp | 20 bp |
|  |  | **Right Primer** | CCGCCAATTATTACGATGCT |  | 191 bp | 20 bp |
| PGSC0003DMG400012887 | M1AZB3 | **Left Primer** | CTTTGGCACCTGAACCTGAG | 205 bp | 8 bp | 20 bp |
|  |  | **Right Primer** | GGGGCTCTTGATCCATGAAA |  | 212 bp | 20 bp |
| PGSC0003DMG400002890 | M0ZT32 | **Left Primer** | CTCTTCTTCGCTTGCCCTTC | 180 bp | 43 bp | 23 bp |
|  |  | **Right Primer** | TGTTGTTGCCGCTAGTTGAA |  | 222 bp | 20 bp |
| PGSC0003DMG400001181 | M0ZL05 | **Left Primer** | CAAAATCGCTCCAGATCCCG | 234 bp | 132 bp | 20 bp |
|  |  | **Right Primer** | TTCCCCTCCGTCGTTATCTC |  | 365 bp | 20 bp |
| PGSC0003DMG400028666 | M1CSN7 | **Left Primer** | CCACCAAGAACAAAGACCCA | 235 bp | 26 bp | 20 bp |
|  |  | **Right Primer** | CCACATCCCAAAACAACCCC |  | 220 bp | 20 bp |
| PGSC0003DMG400008934 | M1AHQ7 | **Left Primer** | CCACCACCTATGCAGCCTAA | 209 bp | 180 bp | 20 bp |
|  |  | **Right Primer** | CGCCAAACATTACCACCCAT |  | 388 bp | 20 bp |
| PGSC0003DMG400023270 | StEIF1α | **Left Primer** | GATGGTCAGACCCGTGAACA | 106 bp | - | 20 bp |
|  |  | **Right Primer** | CCTTGGAGTACTTCGGGGTG |  |  | 20 bp |

**Table S1**. Primer information about selected GATA members and the interacting sequences in potato for qRT-PCR

*Housekeeping gene (StEIF1α) sequence information was received from Tang et al. (2017) study.


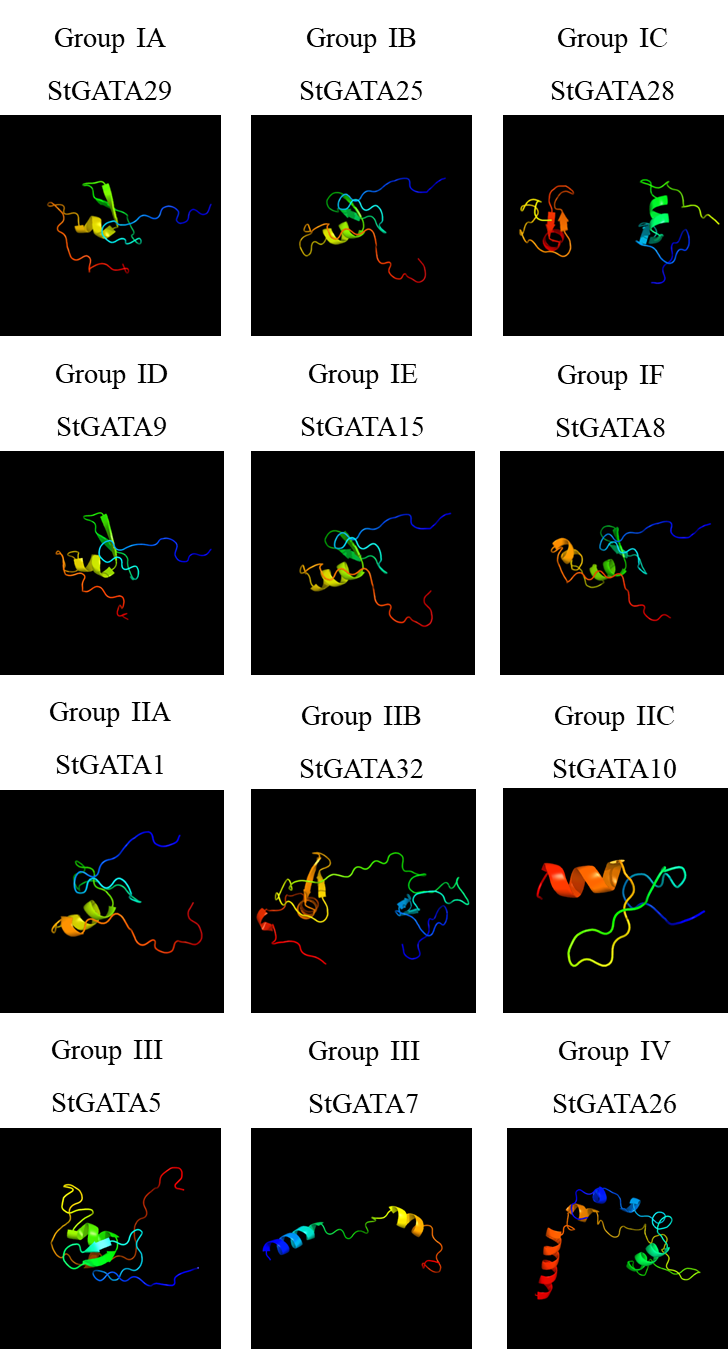


**Figure S1.** Protein models for *StGATA* proteins from different sub-groups in potato


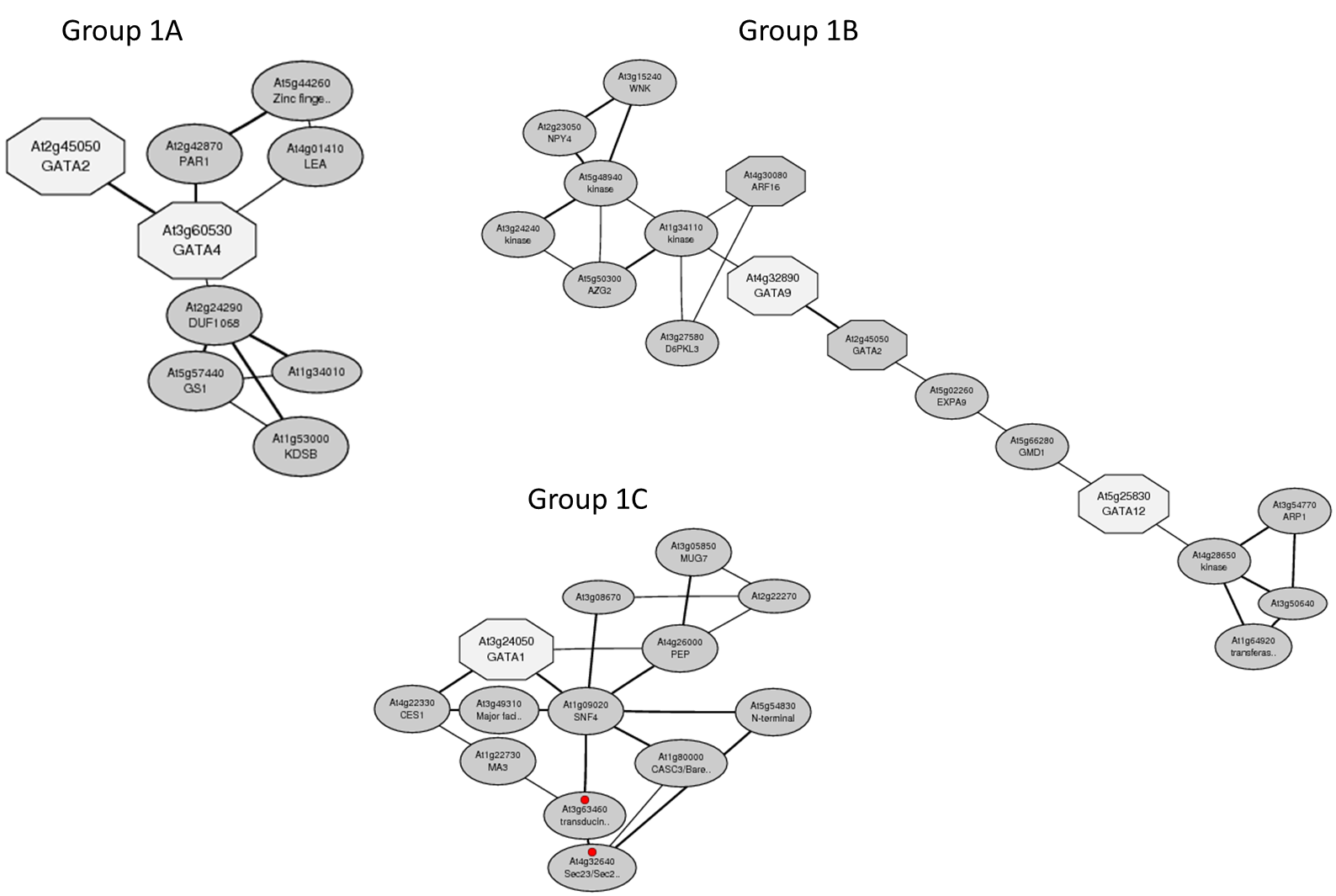


**Figure S2.** Protein-protein interaction analysis of *GATAs* by sub-groups


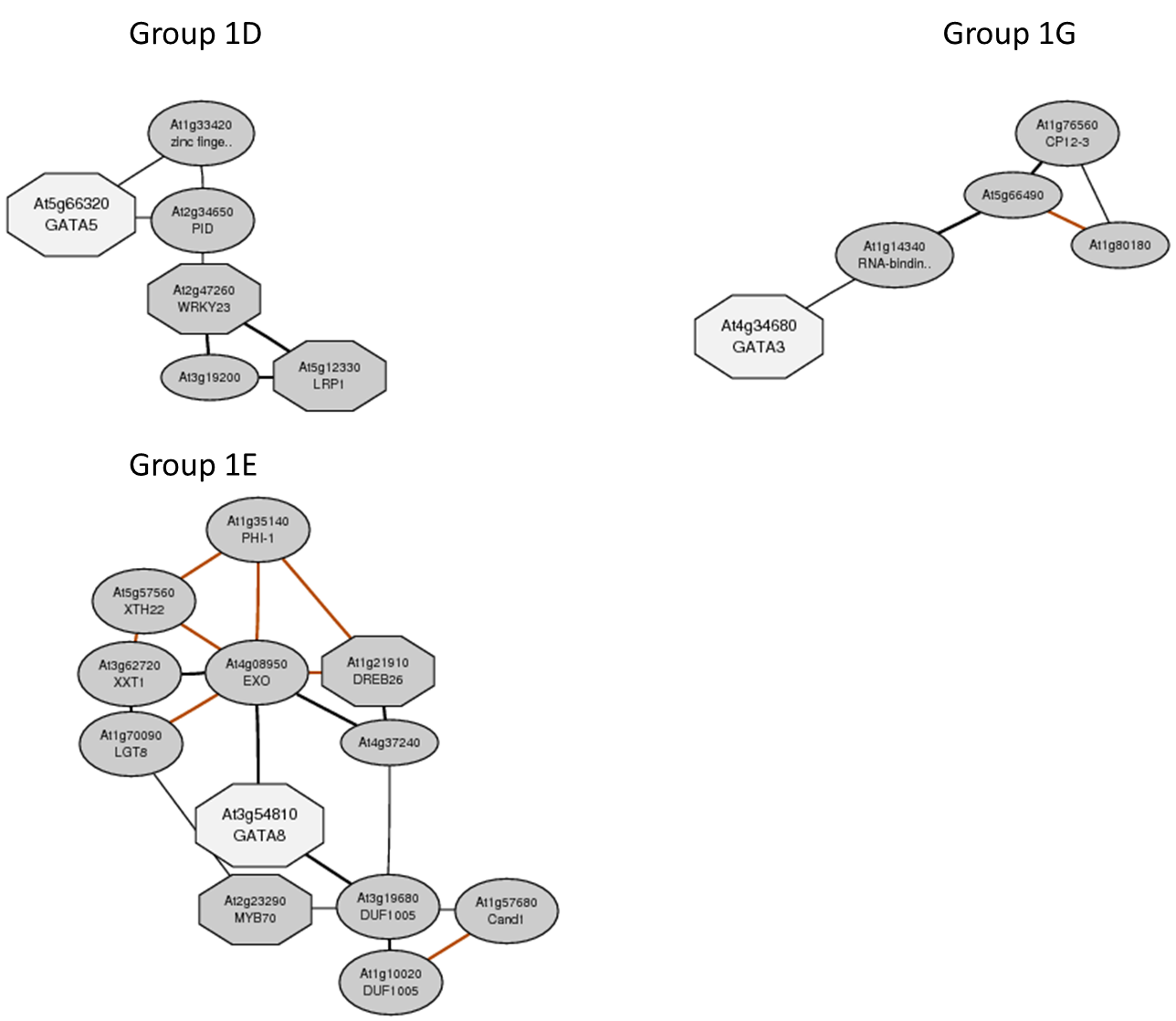


**Figure S2.** Protein-protein interaction analysis of *GATAs* by sub-groups


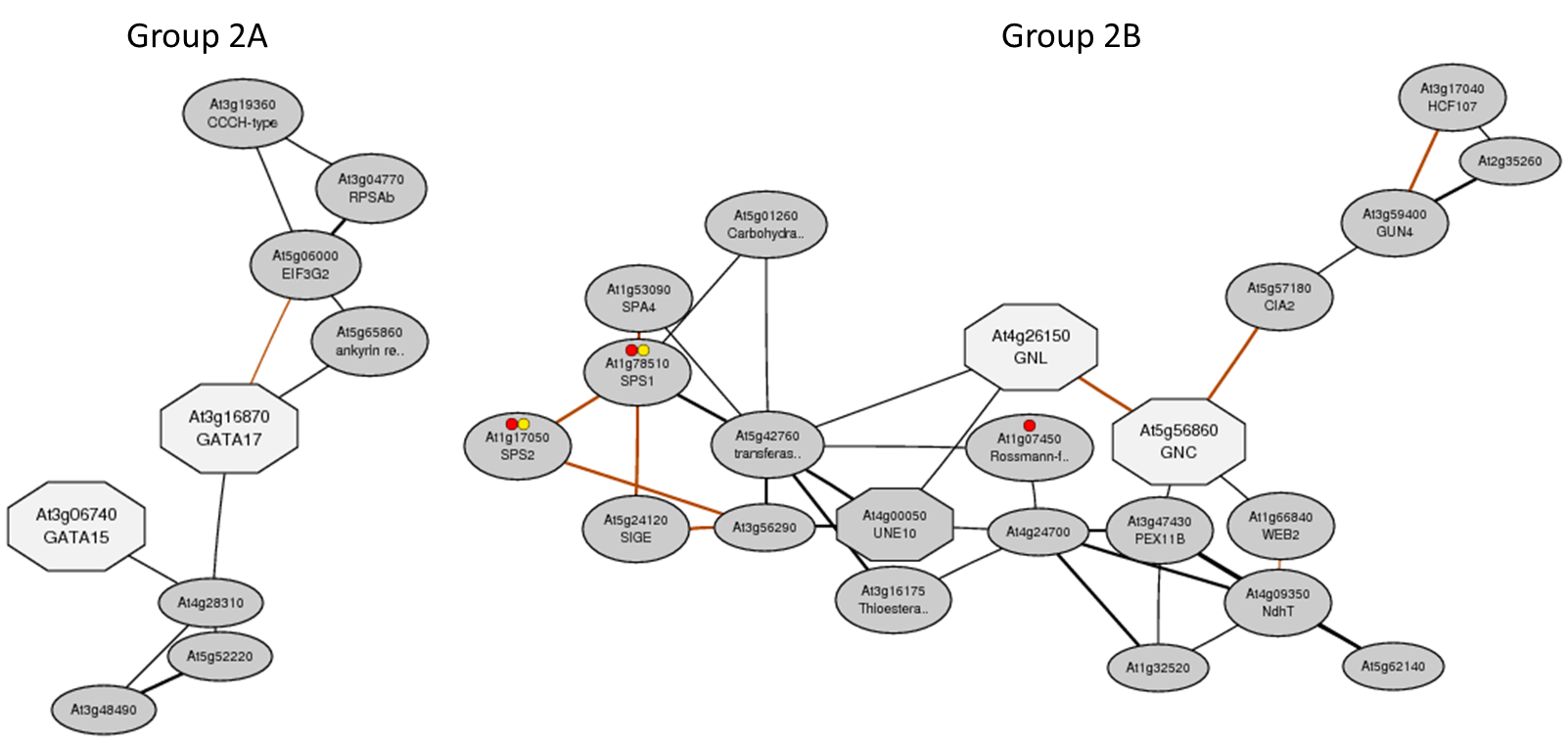


**Figure S2.** Protein-protein interaction analysis of *GATAs* by sub-groups


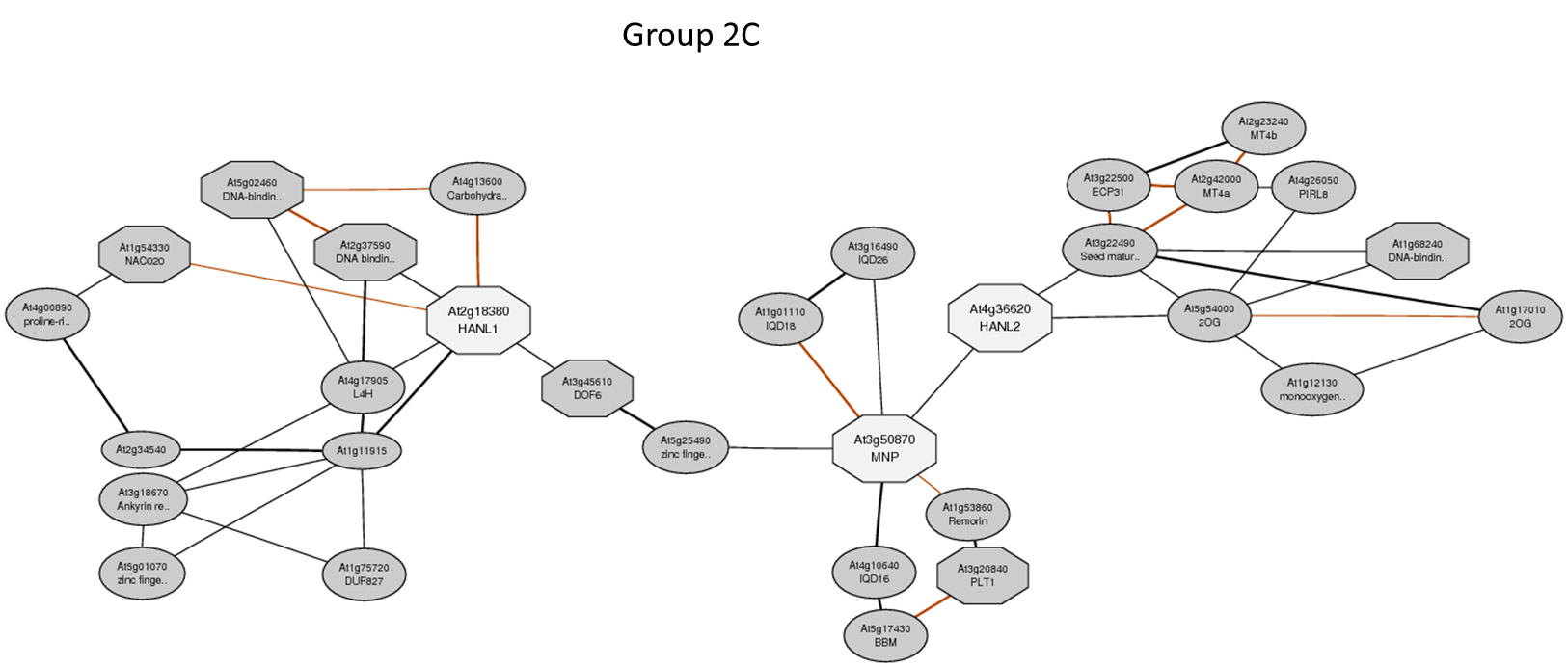


**Figure S2**. Protein-protein interaction analysis of *GATAs* by sub-groups


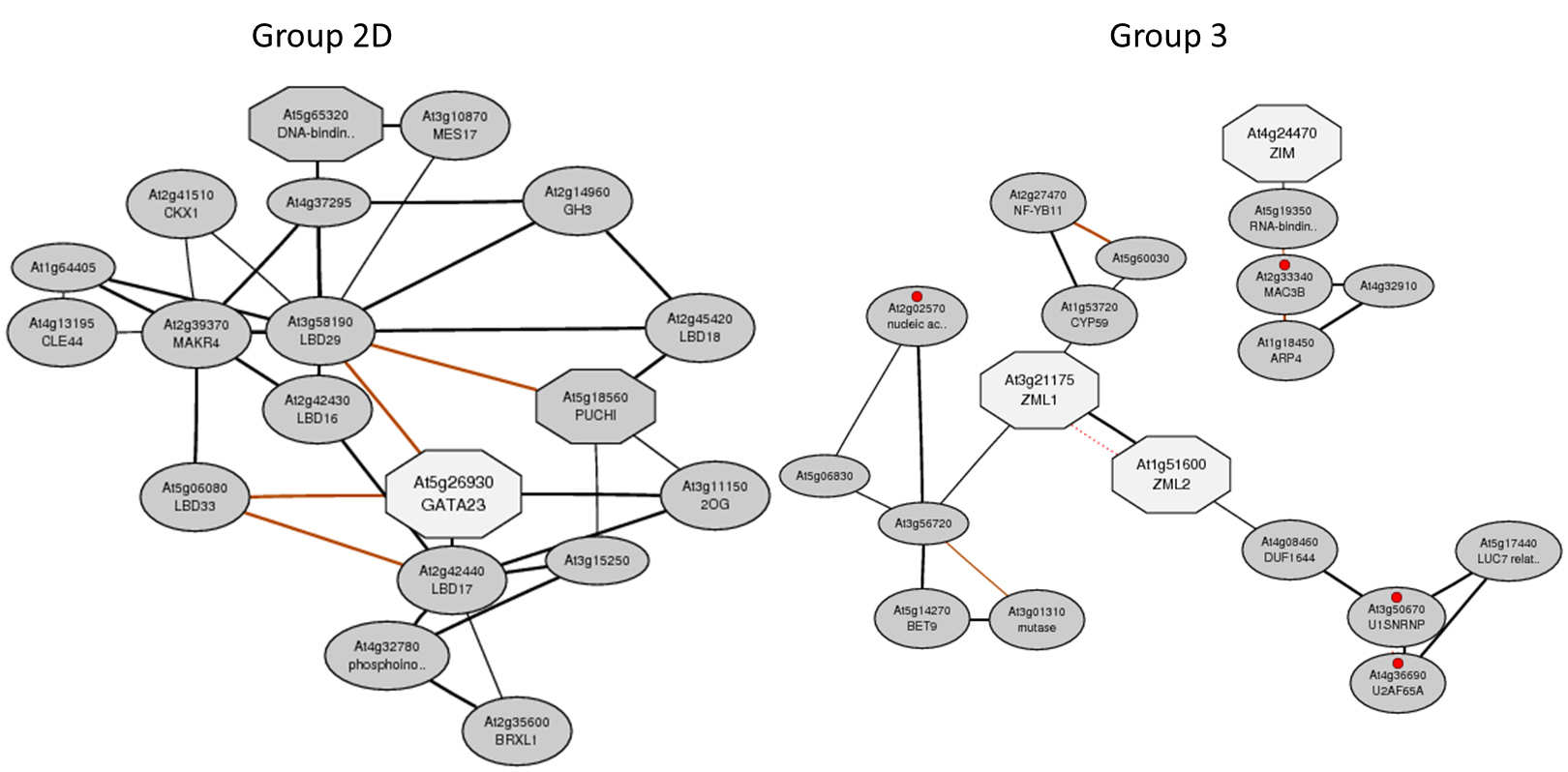


**Figure S2.** Protein-protein interaction analysis of *GATAs* by sub-groups

Group 4


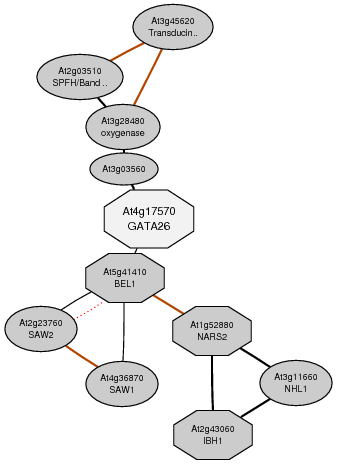


**Figure S2.** Protein-protein interaction analysis of *GATAs* by sub-groups


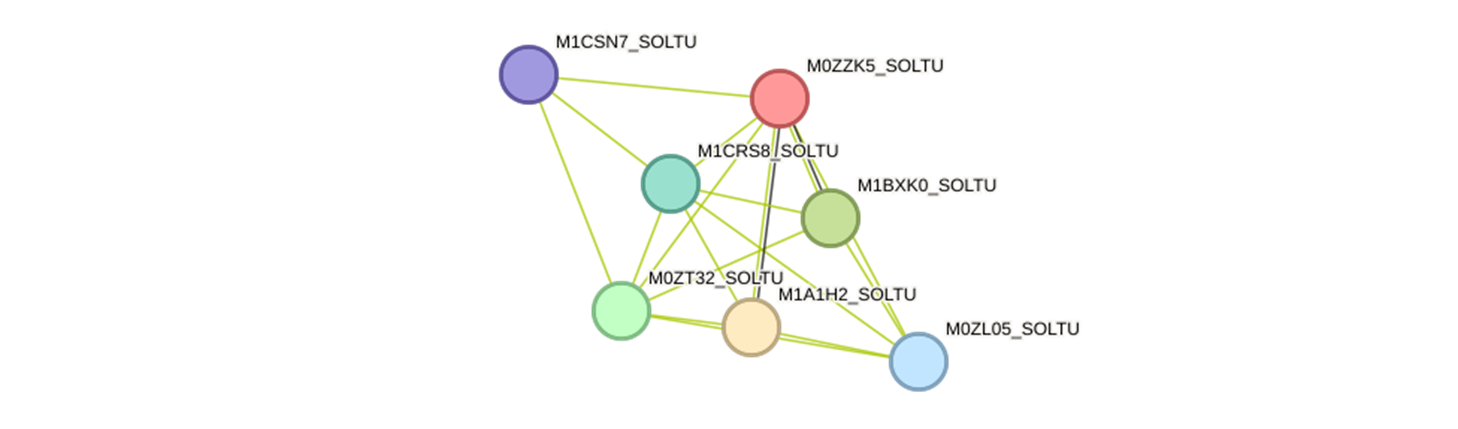

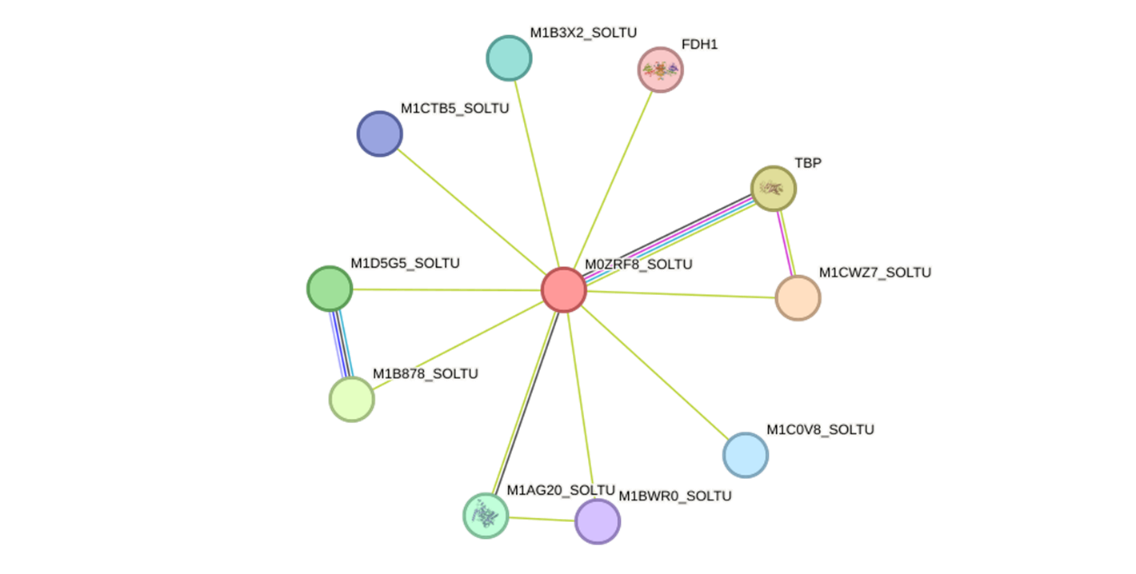

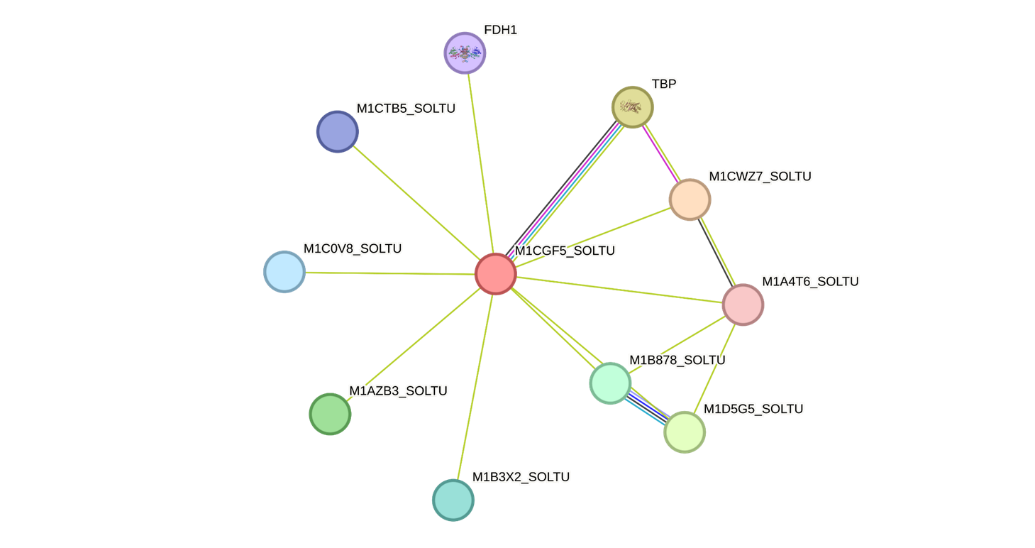
a) b) c)


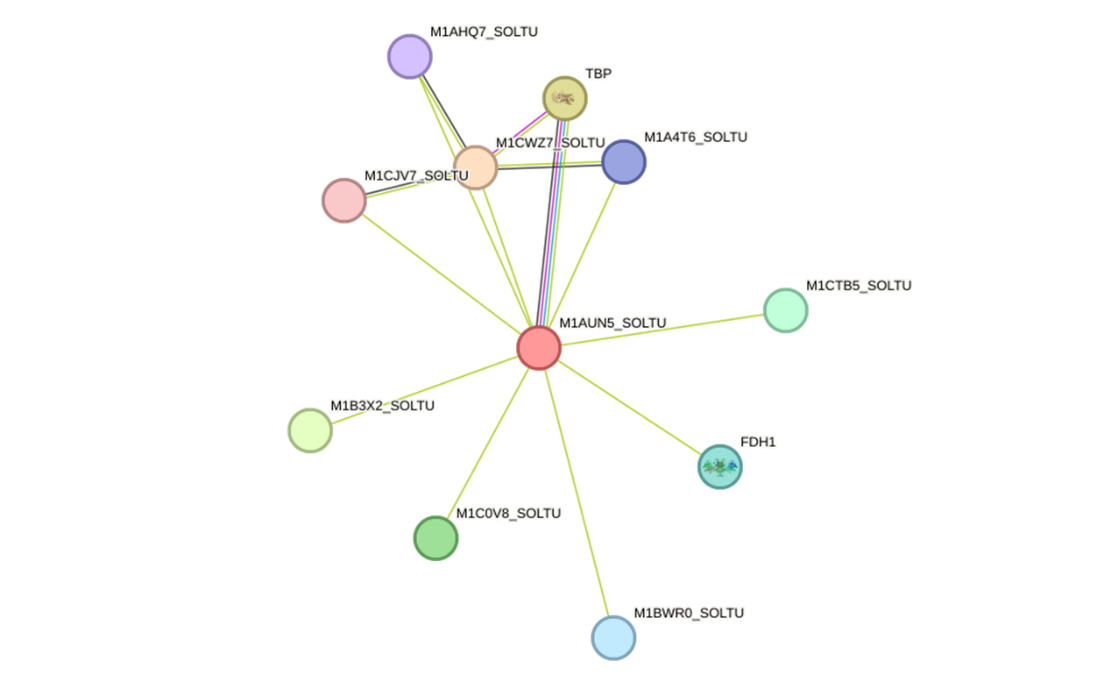


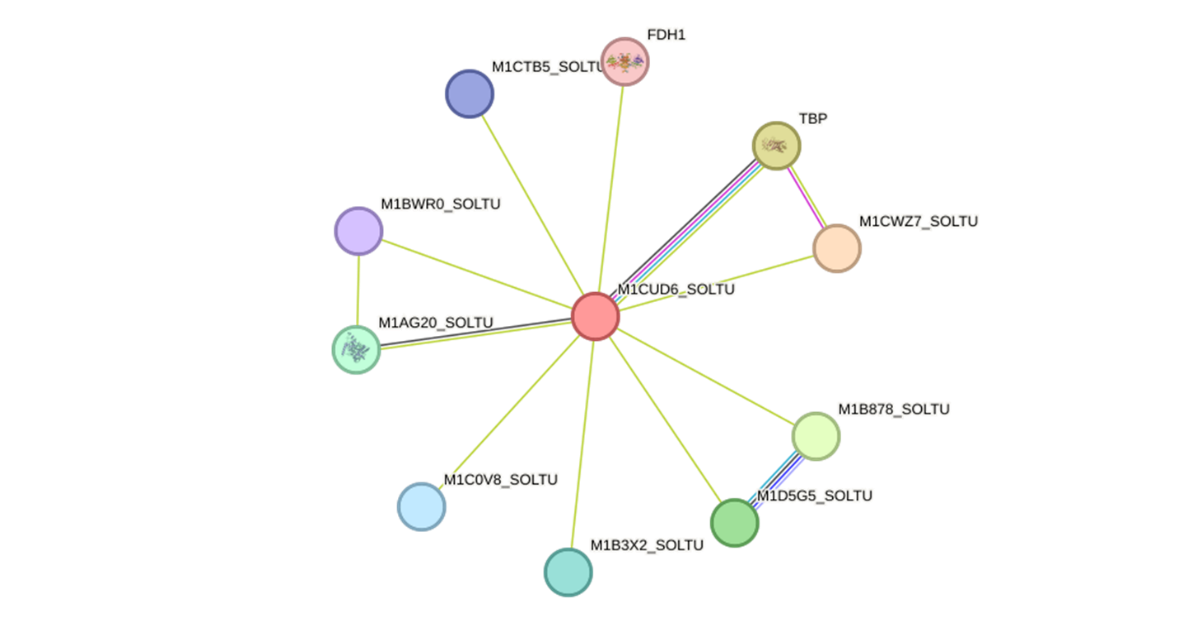
d) e)

**Figure S3.** Protein-protein interaction analysis of selected *StGATAs*, a) *StGATA3*, b) *StGATA15*, c) *StGATA24*, d) *StGATA25*, and e) *StGATA29*


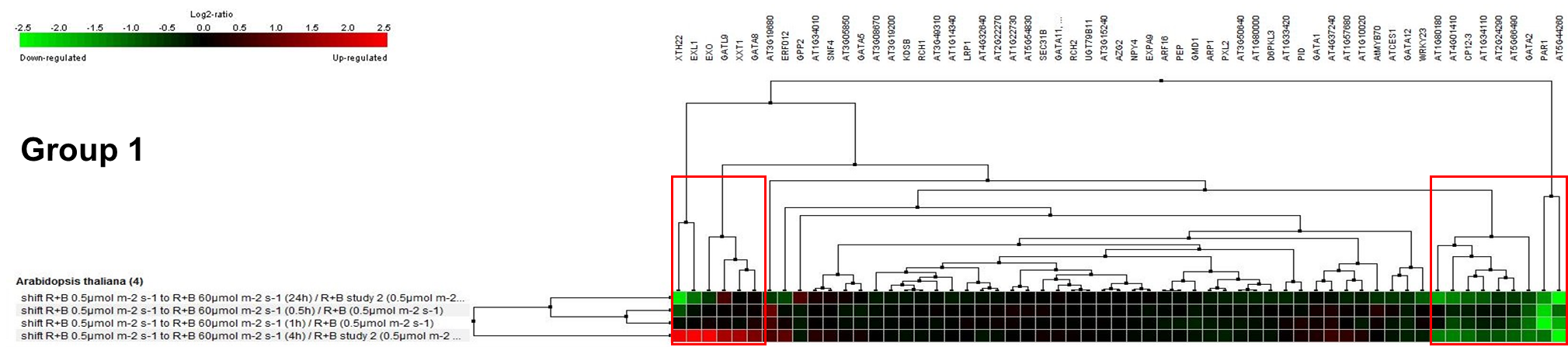

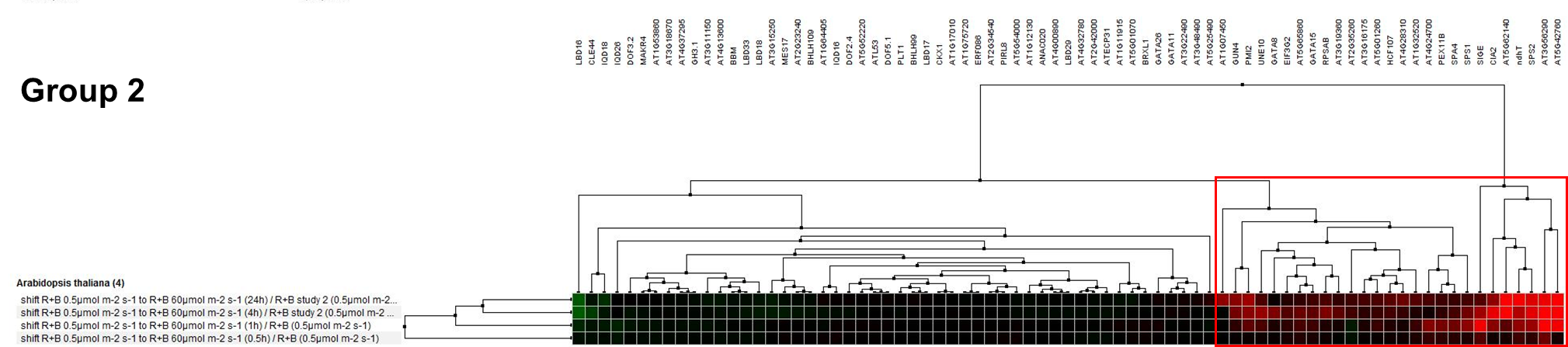

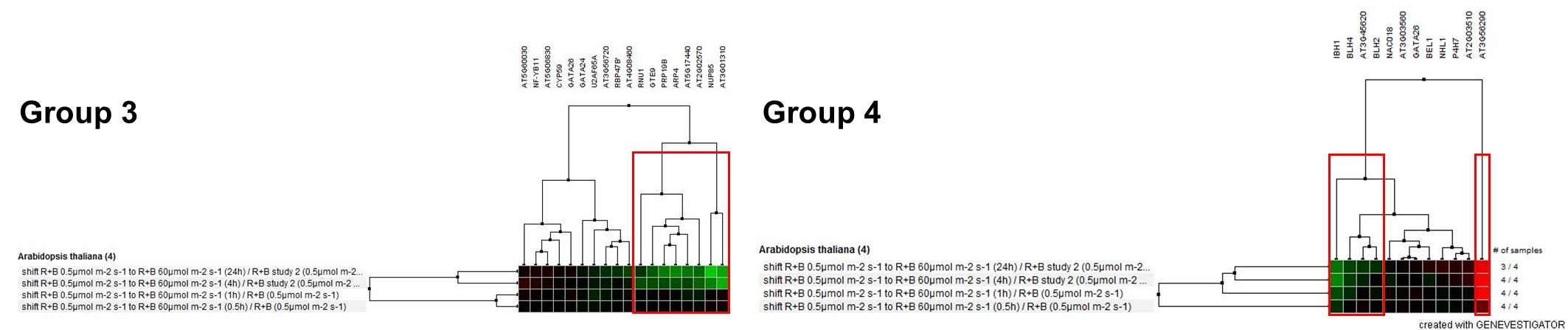


**Figure S4.** Hierarchical clustering of co-expression network genes under high red/blue light. Expression patterns of all the genes in different groups of co-expression network drawn by genes interacting with *AtGATA*s were determined in the microarray experiment on high red/blue light for different durations (GEO series of GSE31587). Red boxes indicate the genes with the most significant high or low expression that were used for further analyses.


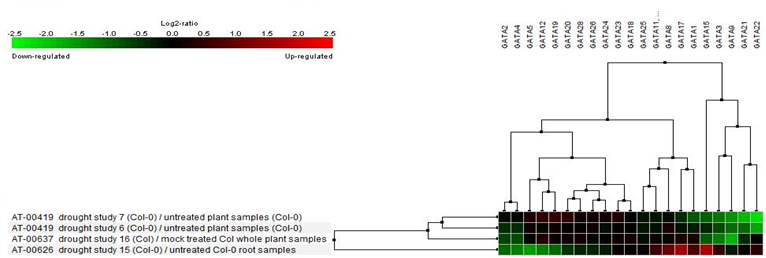

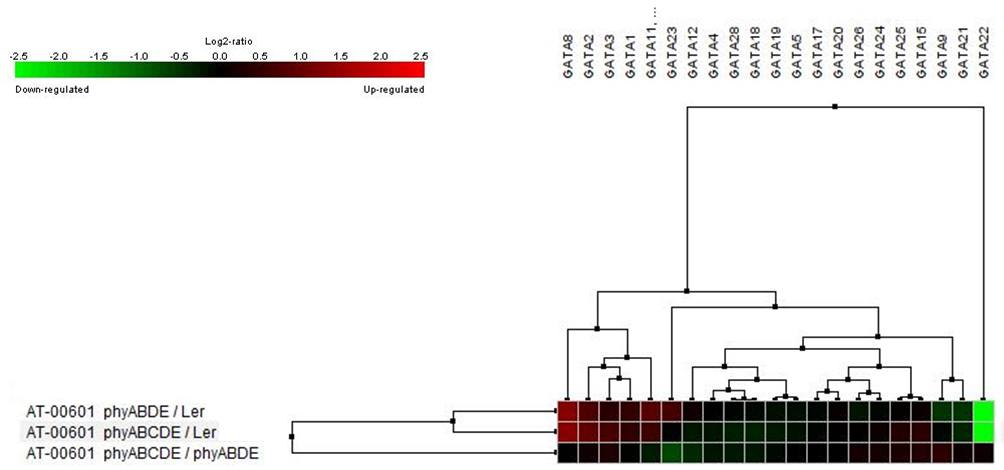


a)

b)

**Figure S5.** In silico expression pattern of Arabidopsis *GATA*s under high blue- and red-light intensities as well as light-receptor mutants. a) Heat map of AtGATAs under high blue/red-light intensities. b) Heat map of AtGATAs in light-receptor mutants.


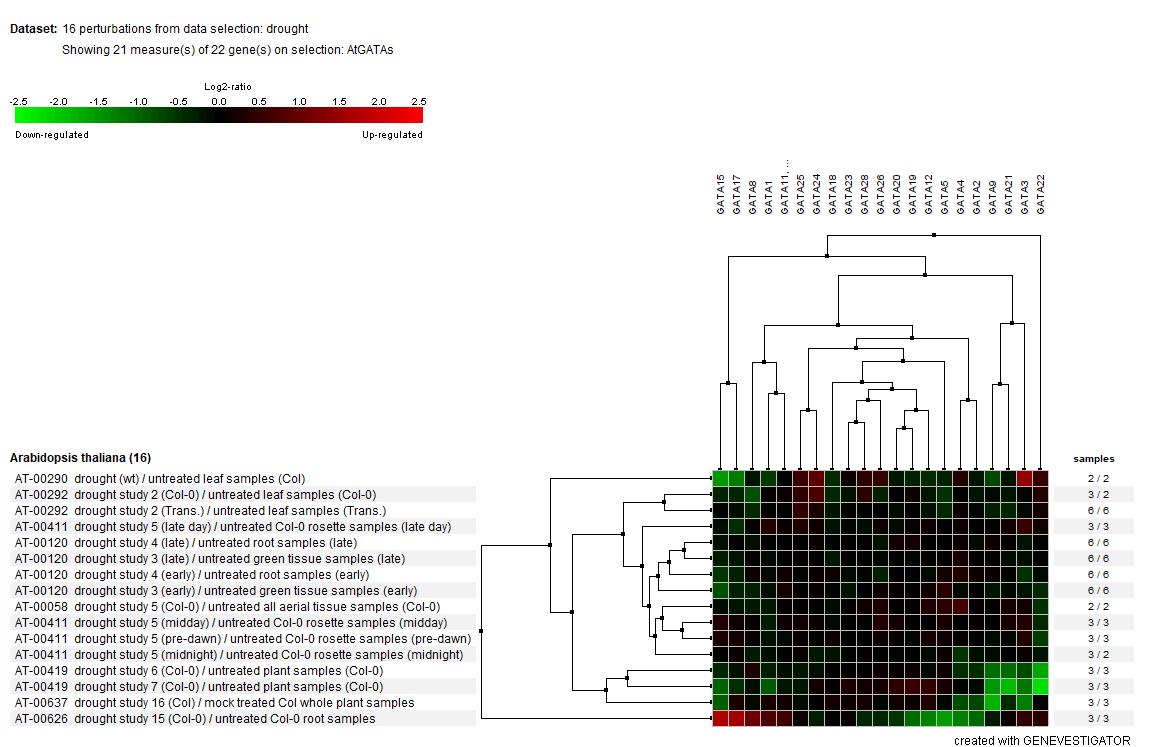

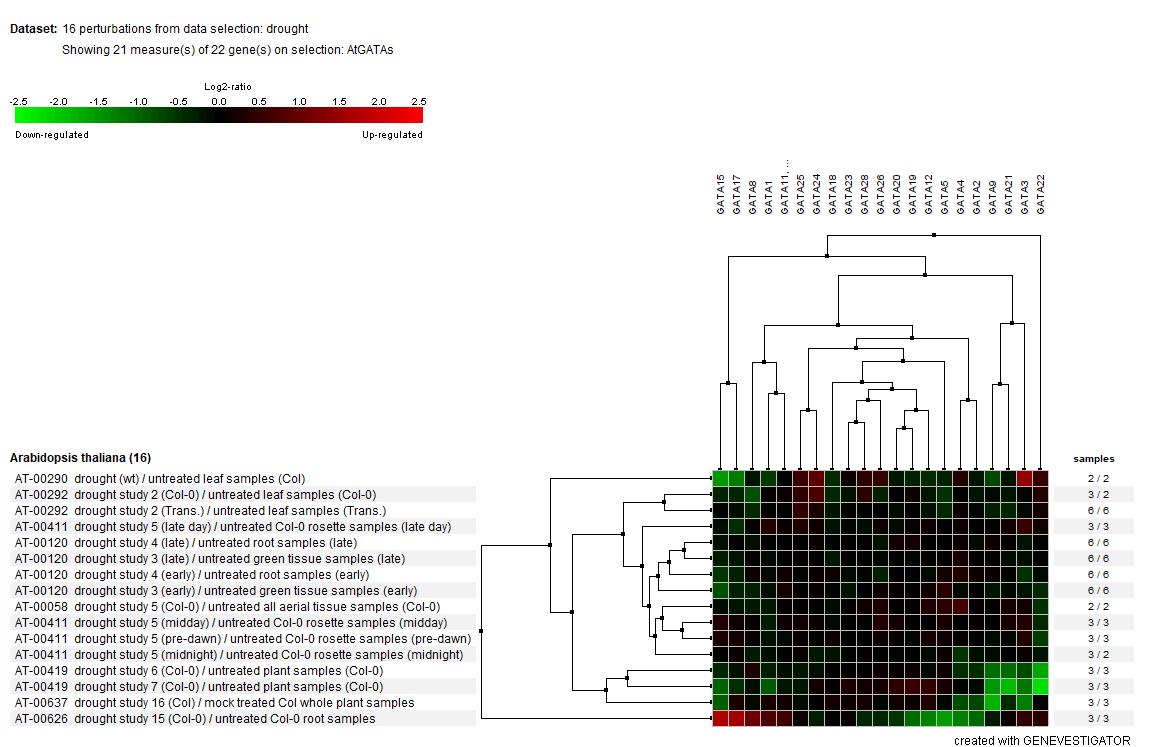


**Figure S6.** In silico expression pattern of Arabidopsis *GATA*s under drought. Heat map of *AtGATA*s under differnet drought experiements were drawn by Genevestigator. Hierarchical clustering was completed by Manhattan distance with optimal leaf-ordering of genes and conditions.

0,00

10,00

20,00

30,00

40,00

50,00

60,00

70,00

80,00

WC

WD

RC

RD

BC

BD

PC

PD

0,00

10,00

20,00

30,00

40,00

50,00

60,00

70,00

80,00

90,00

WC

WD

RC

RD

BC

BD

PC

PD

0,00

50,00

100,00

150,00

200,00

250,00

WC

WD

RC

RD

BC

BD

PC

PD

a)

b)

**Agria**

**Sante**

a

d

b

f

b

f

f

c

e

bc

de

cd

e

a

a

de

a

a

b

b

c

a

c

c

0,00

50,00

100,00

150,00

200,00

250,00

300,00

350,00

WC

WD

RC

RD

BC

BD

PC

PD

b

cd

a

d

c

b

cd

d

**Suppl. Figure 12.** Physiological responses of two potato cultivars to a combination of drought and light. a) Shoot length. b) Root length.

**Figure S7**. Physiological responses of two potato cultivars to a combination of drought and light. **a)** Shoot length (mm). **b)** Root length (mm) (C: control, D: drought, W: white, R: red, B: blue, P: purple).

**Table S2.** Annotation of selected *StGATA* interacting proteins

| **Query** | **Interacting Proteins (from STRING)** | | | | | | | |
| --- | --- | --- | --- | --- | --- | --- | --- | --- |
|  | **STRING #** | **Spud DB Accession #** | **Annotation in STRING** | **NCBI Accession #** | **Annotation in NCBI** | ***cis*-element function** | **GATA element in promoter** |  |
| StGATA3 | M1CWZ7_SOLTU | PGSC0003DMP400045152 | Ethylene-responsive transcriptional coactivator | NW_006239061.1 | PREDICTED: Solanum tuberosum multiprotein-bridging factor 1c (LOC102594575), mRNA | light | - |  |
|  | TBP | No hit | TATA-box-binding protein | NW_006239213.1 | TATA-box-binding protein-like | light+drought | - |  |
|  | ***M1AZB3_SOLTU*** | ***PGSC0003DMG400012887*** | ***Cyclin; Belongs to the cyclin family*** | ***NW_006239037.1*** | ***cyclin-P3-1*** | ***light*** | ***+*** |  |
|  | M1C0V8_SOLTU | PGSC0003DMG400022265 | Vesicle transport protein | NW_006239000.1 | protein transport protein SFT2-like | light |  |  |
|  | M1CTB5_SOLTU | PGSC0003DMG400028831 | Vesicle transport protein | NW_006239064.1 | protein transport protein SFT2-like | light | - |  |
|  | Q07511, FDH1 | No hit | Formate dehydrogenase, mitochondrial | NW_006238930.1 | mitochondrial formate dehydrogenase precursor | light+drought | - |  |
|  | M1A4T6_SOLTU | PGSC0003DMG400005746 | Uncharacterized protein | NW_006238952.1 | splicing factor 3B subunit 4-like | light+drought | - |  |
| StGATA15 | M1CWZ7_SOLTU | PGSC0003DMG400029773 | HTH cro/C1-type domain-containing protein | NW_006239061.1 | multiprotein-bridging factor 1c^1^ | light | - |  |
|  | TBP | None | TATA-box-binding protein | NW_006239213.1 | TATA-box-binding protein-like | light+drought | - |  |
|  | M1B878_SOLTU | PGSC0003DMG400015233 | Uncharacterized protein | no info | no info | no info | - |  |
|  | M1D5G5_SOLTU | PGSC0003DMG400032200 | Uncharacterized protein | no info | no info | no info | - |  |
|  | M1AG20_SOLTU | PGSC0003DMG400008545 | Photolyase/cryptochrome alpha/beta domain-containing protein | NW_006239241.1 | cryptochrome-1-like | light+drought | - |  |
|  | M1B3X2_SOLTU | PGSC0003DMG400014119 | Vesicle transport protein | no info | no info | no info | - |  |
|  | M1C0V8_SOLTU | PGSC0003DMG400022265 | Vesicle transport protein | NW_006239000.1 | protein transport protein SFT2-like | light | - |  |
|  | M1CTB5_SOLTU | PGSC0003DMG400028831 | Vesicle transport protein | NW_006239064.1 | protein transport protein SFT2-like | light+drought | - |  |
|  | M1BWR0_SOLTU | PGSC0003DMG401021239 | C2H2-type domain-containing protein | no info | no info | no info | - |  |
|  | FDH1 | None | Formate dehydrogenase, mitochondrial | NW_006238930.1 | mitochondrial formate dehydrogenase precursor | light+drought | - |  |
| StGATA24 | M1A1H2_SOLTU | PGSC0003DMG400004921 | Uncharacterized protein | no info | no info | no info | - |  |
|  | M1BXK0_SOLTU | PGSC0003DMG402021429 | Uncharacterized protein | no info | no info | no info | - |  |
|  | ***M0ZT32_SOLTU*** | ***PGSC0003DMG400002890*** | ***SPX domain-containing protein*** | ***NW_006238988.1*** | ***SPX domain-containing protein 1*** | ***light*** | ***+*** |  |
|  | M1CRS8_SOLTU | PGSC0003DMG401028489 | Magnesium transporter | NW_006239140.1 | magnesium transporter MRS2-1 | light+drought | - |  |
|  | ***M0ZL05_SOLTU*** | ***PGSC0003DMG400001181*** | ***MCU domain-containing protein*** | ***NW_006238985.1*** | ***calcium uniporter protein 2, mitochondrial*** | ***light+drought*** | ***+*** |  |
|  | ***M1CSN7_SOLTU*** | ***PGSC0003DMG400028666*** | ***Protein kinase domain-containing protein*** | ***NW_006239054.1*** | ***mitogen-activated protein kinase kinase kinase YODA*** | ***light+drought*** | ***+*** |  |
| StGATA25 | M1CWZ7_SOLTU | PGSC0003DMG400029773 | HTH cro/C1-type domain-containing protein | NW_006239061.1 | multiprotein-bridging factor 1c | light | - |  |
|  | TBP | None | TATA-box-binding protein | NW_006239213.1 | TATA-box-binding protein-like | light+drought | - |  |
|  | M1B878_SOLTU | PGSC0003DMG400015233 | Uncharacterized protein | no info | no info | no info | - |  |
|  | M1D5G5_SOLTU | PGSC0003DMG400032200 | Uncharacterized protein | no info | no info | no info | - |  |
|  | M1AG20_SOLTU | PGSC0003DMG400008545 | Photolyase/cryptochrome alpha/beta domain-containing protein | NW_006239241.1 | cryptochrome-1-like | Light | - |  |
|  | M1B3X2_SOLTU | PGSC0003DMG400014119 | Vesicle transport protein | no info | no info | no info | - |  |
|  | M1C0V8_SOLTU | PGSC0003DMG400022265 | Vesicle transport protein | NW_006239000.1 | protein transport protein SFT2-like | Light | - |  |
|  | M1CTB5_SOLTU | PGSC0003DMG400028831 | Vesicle transport protein | NW_006239064.1 | protein transport protein SFT2-like | light+drought | - |  |
|  | M1BWR0_SOLTU | PGSC0003DMG401021239 | C2H2-type domain-containing protein | no info | no info | no info | - |  |
|  | FDH1 | None | Formate dehydrogenase, mitochondrial | NW_006238930.1 | mitochondrial formate dehydrogenase precursor | light drought | - |  |
| StGATA29 | M1CWZ7_SOLTU | PGSC0003DMG400029773 | HTH cro/C1-type domain-containing protein | NW_006239061.1 | multiprotein-bridging factor 1c | Light | - |  |
|  | TBP | None | TATA-box-binding protein | NW_006239213.1 | TATA-box-binding protein-like | Light+drought | - |  |
|  | M1B3X2_SOLTU | None | Vesicle transport protein | None | None | None | - |  |
|  | M1C0V8_SOLTU | PGSC0003DMG400022265 | Vesicle transport protein | NW_006239000.1 | protein transport protein SFT2-like | Light+drought | - |  |
|  | M1CTB5_SOLTU | PGSC0003DMG400028831 | Vesicle transport protein | NW_006239064.1 | protein transport protein SFT2-like | Light+drought | - |  |
|  | FDH1 | None | Formate dehydrogenase, mitochondrial | NW_006238930.1 | mitochondrial formate dehydrogenase precursor | Light+drought | - |  |
|  | M1BWR0_SOLTU | PGSC0003DMG401021239 | C2H2-type domain-containing protein | None | None | None | - |  |
|  | M1A4T6_SOLTU | PGSC0003DMG400005746 | Uncharacterized protein | NW_006238952.1 | splicing factor 3B subunit 4-like | Light+drought | - |  |
|  | ***M1AHQ7_SOLTU*** | ***PGSC0003DMG400008934*** | ***Uncharacterized protein*** | ***NW_006238947.1*** | ***splicing factor 3B subunit 4-like*** | ***Light+drought*** | ***+*** |  |
|  | M1CJV7_SOLTU | PGSC0003DMG400026862 | RRM domain-containing protein | NW_006239137.1 | splicing factor 3B subunit 4 | Light | - |  |
