# Supplementary material for: Genome‐wide characterization and expression analysis of GATA transcription factors under combination of light wavelengths and drought stress in potato
Source: Plant Direct. 2024 Apr 24;8(4):e569. doi: 10.1002/pld3.569 (PMC11042883; doi:10.1002/pld3.569)
Supplement: Supplementary file 1 — Data S1. Peer Review. [file PLD3-8-e569-s001.pdf]

Plant Direct

Open Access

GATA Transcription Factor Family in Potato

Emre Aksoy[1], Caner Yavuz[2]*, Ayten Kübra Yagiz[2], Necdet Mehmet Unel[3,4], Mehmet Cengiz Baloğlu[3,5]

DECISION LETTER- ROUND 1

November 11, 2023

Dr. Caner Yavuz
Niğde Ömer Halisdemir University
Merkez
Niğde, N/A 51240
Turkey

RE: Genome-wide Characterization and Expression Analysis of GATA Transcription Factors Under Combination of Light Wavelengths and Drought Stress in Potato

Dear Dr. Yavuz:

Thank you for submitting to Plant Direct. All required reviews have been returned and we have now finished our evaluation of your manuscript. In light of the reviewers' and editor's comments, further revisions are needed before the paper can be accepted for publication in Plant Direct.

Please view the editors' and reviewers' comments below and use their suggestions as a guide while you work on your revision.

When uploading the revised version of this article, please be sure to include the following:

-A word document that contains your response to the reviewers. You should respond to each reviewer comment and note the changes made to the manuscript. If you do not agree with a reviewer's comment and choose not to make a suggested revision, please explain why. Please try to provide as complete an answer as possible to each reviewer's criticisms.
-A tracked changes document with each change highlighted
- A clean version of the latest version of the manuscript

Show the role of StGATA3 under drought stress and the light response mechanisms in potato or any heterologous system.

To upload your revision, please click the link below.
https://plantdirect.msubmit.net/cgi-bin/main.plex?el=A6Lr1cz5A6oQE6I7A9ftdqxJMctyTwcOe9yOjn8xxQZ

In order to provide as timely a service as possible, we ask that your revision is resubmitted within three months after receipt of this request. If an extension is needed, please send a request, along with a brief explanation, to the editorial office at plantdirect@wiley.com .

Please note that, in addition to publishing reviewer comments, the author's responses to review comments will also be published alongside the final version of the paper. If you would not like the author's responses to be published, please contact the editorial office at plantdirect@wiley.com .

Thank you very much for giving us an opportunity to review your work. I look forward to receiving the next version.

Sincerely,

Divya Mishra

Esther van der Knaap

Editor, Plant Direct

-- Editor comments:
Show the role of StGATA3 under drought stress and the light response mechanisms in potato or any heterologous system.

-- Reviewer comments:
Reviewer #1:

I carefully read the research paper entitled "Genome-wide Characterization and Expression Analysis of GATA Transcription Factors Under Combination of Light Wavelengths and Drought Stress in Potato" to gain a comprehensive understanding of its content. Using various in silico tools, GATA TFs were identified throughout the potato genome in this study. The in silico study revealed that a comprehensive annotation process had been performed. The entire text, particularly the introduction and discussion sections, is composed with utmost scientific precision and organization, but it is recommended that authors make minor English corrections before publishing. Several previous studies have established that GATAs are critical for light signaling. Still, the cis-regulatory elements identified in this study have shown that TFs might also play significant roles in abiotic stress mechanisms, such as drought. With the subsequent in silico protein-protein interaction study, it was seen that the proteins in Group I, for example, may be responsible for both light and abiotic stress responses. Given that, I believe it is crucial to scrutinize both single and combined stress in sensitive and tolerant potato varieties for the authenticity of the research. Considering the significant value of the findings in this study, particularly with regard to combined stress, I believe it should be accepted without requiring any further scientific revisions.

Reviewer #2:

The study titled "Genome-wide Characterization and Expression Analysis of GATA Transcription Factors Under Combination of Light Wavelengths and Drought Stress in Potato" has covered in silico GATA TFs annotation and the response to light, drought, and combined stress of several selected GATA TFs. The study presents new insights, especially about the expression levels of GATA genes under combined treatment of drought and different light wavelengths. GATA TFs are mostly known for their function in light response; however, the emphasis on the role of drought response is another contribution of this study to the existing literature on GATA TFs. I believe the study is well-established with various bioinformatics analyses and RT-qPCR. However, there are some minor revisions that need to be taken care of.

The introduction is written quite well; however, the following sentences need corrections and clarifications;

· The sentence beginning with "It is worth noting that plants exhibit ...." Is difficult to understand. Please make it clear. (Lines 71-73)

· Please check and add recent publications to strengthen the hypothesis for the interconnection between light and drought mechanisms. (Lines 73-75)

· Correct "moso bamboo" as "Moso bamboo". (Line 82)

· Correct "salinity toleranca" as "salinity tolerance". (Line 95)

The methodology was explained thoroughly, but a few points require further elucidation;

· Change "internal control" to "housekeeping gene". (Line 207)

· Add the sequence information of the housekeeping gene to Table S1 in the supplementary file. (Line 207)

· Correct "StgGATA" with "StGATA". (Line 225)

· Can you write the number of StGATAs present in chromosome I in a sentence? (Lines 227-228)

· Please make the sentence clear starting with "Unfortunately, grouping according to gene structure did...". What do you try to explain? (Lines 234-235)

· Can you please very briefly mention the details for the function of AtGATA03 and AtGATA29 in the discussion part? (Lines 240)

· Please add numeric data for the phenotypic observations. (Line 331)

· You mentioned that "Sante was relatively tolerant to light". Some information is missing here. Which particular light? (Line 333)

· Correct "25-folds" with "25-fold". (Line 342)

· The sentence that starts with "Although our sub-groups did not exhibit conserved gene structure patterns" is not clear. Please make it clear. (Lines 374-375)

· Is there a typo in the sentence that starts with "Unlike Group IIB, Group II comparably...."? I think there is a mistake here. (Line 446)

· Please give units to shoot and root lengths in Figure S6 in the supplementary file.

The results, discussion, and conclusion sections are well-written without any errors.

DECISION LETTER- ROUND 2

January 31, 2024

Dr. Caner Yavuz
Niğde Ömer Halisdemir University
Merkez
Niğde, N/A 51240
Turkey

MSID: 2023-01295R1
MS TITLE: Genome-wide Characterization and Expression Analysis of GATA Transcription Factors Under Combination of Light Wavelengths and Drought Stress in Potato

Dear Dr. Caner Yavuz:

I am pleased to inform you that your manuscript "Genome-wide Characterization and Expression Analysis of GATA Transcription Factors Under Combination of Light Wavelengths and Drought Stress in Potato" has been accepted for publication in Plant Direct.

Your article will appear online in the next available issue of Plant Direct. To ensure your article gets published as quickly as possible, please pay attention to the steps detailed below. We have found that most of the delays happen at this stage, especially at the payment stage, so please respond as quickly as possible when prompted.

License Agreement: Once your article has been accepted it will move to Production and undergo admin and file checking - you may receive an email with any queries we have at this stage. When all required items are received by the publisher and queries resolved, the corresponding author will receive an email from Wiley's Author Services system which will ask them to log on at https://authorservices.wiley.com/bauthor and will present them with the appropriate license for completion. Your article cannot be published until both the signed license agreement and payment of the article fee have been received.

Payment of the Open Access Article Publication Fee: All articles published in Plant Direct are fully open access: immediately and freely available to read, download and share. Plant Direct charges a publication fee to cover the publication costs. The corresponding author for this manuscript should have already received a quote from the payments team (cs-openaccess@wiley.com) with the

estimated article publication fee; please email cs-openaccess@wiley.com if this has not been received. The corresponding author should log on to the Wiley Author Services site, where the publication fee can be paid by credit card or an invoice. Pro Forma can also be requested. Payment of the publication charge must be received before the article will be published online.

Proofs: You will have the opportunity to look over your paper once more when you receive the author proofs for your article. The proofs will be with you in approximately two weeks. Please note that, in addition to publishing reviewer comments, the author's responses to review comments will also be published alongside the final version of the paper. If you would not like the author's responses to be published, please contact the editorial office at plantdirect@wiley.com .

Promotion of your article: You can help your research get the attention it deserves! Check out Wiley's free Promotion Guide for best-practice recommendations for promoting your work at www.wileyauthors.com/eeo/guide. And learn more about Wiley Editing Services which offers professional video, design, and writing services to create shareable video abstracts, infographics, conference posters, lay summaries, and research news stories for your research at www.wileyauthors.com/eeo/promotion.

Thank you again for your contribution to Plant Direct. If you have any questions, feel free to contact the editorial office at plantdirect@wiley.com .

Sincerely,

Divya Mishra

Esther van der Knaap

Editor, Plant Direct

-- Editor comments

-- Reviewer comments:

AUTHORS' RESPONSE

To the Editor

Query: Show the role of StGATA3 under drought stress and the light response mechanisms in potato or any heterologous system.

Corresponding author: We would like to thank the Editor and Reviewers for taking your time to review our manuscript. We have a limited budget and time to address the additional request about heterologous StGATA3 expression. Owing to customs and other circumstances, it would take several months for the necessary materials and chemicals to reach us on time, even if we had the budget.

Before submitting our article to your journal, based on our study findings, we had already planned to conduct the functional characterization of the genes for which the expression levels were checked with qPCR, particularly for combined stress in potato. We are currently working on a project proposal that meets budget constraints.

Instead, in order to meet and respond to the Editor's current revision request in an affordable and time effective manner, the authors agree on conducting further experimentation for downstream protein–protein interaction analysis. Initially, the protein interaction network analysis was revealed for six StGATAs and the interacting partners were checked for the GATA motif. Those with the GATA motif were further screened for expression changes under light, drought, and combined stress conditions using qPCR. The authors accept that the findings are still preliminary; however, especially regarding the annotation in literature specifying a role during stomatal development and activity by a phytochrome-mediated approach reinforces the role of StGATA proteins during light, drought, and combined stress. Especially StGATA24 interacting protein YODA is well studied in the literature, and previous findings endorse our current findings.

We kindly ask Editor to consider this situation when re-evaluating our article. The additions are highlighted in yellow (Materials and Methods: 212-215, Results: Lines 367-398, Discussion: 546-587; 591-593). Relevant citations were made in the respective places in the References section.

Respond to Reviewer 1

Query: I carefully read the research paper entitled "Genome-wide Characterization and Expression Analysis of GATA Transcription Factors Under Combination of Light Wavelengths and Drought Stress in Potato" to gain a comprehensive understanding of its content. Using various in silico tools, GATA TFs were identified throughout the potato genome in this study. The in silico study revealed that a comprehensive annotation process had been performed. The entire text, particularly the introduction and discussion sections, is composed with utmost scientific precision and organization, but it is recommended that authors make minor English corrections before publishing. Several previous studies have established that GATAs are critical for light signaling. Still, the cis-regulatory elements identified in this study have shown that TFs might also play significant roles in abiotic stress mechanisms, such as drought. With the subsequent in silico protein-protein interaction study, it was seen that the proteins in Group I, for example, may be responsible for both light and abiotic stress responses. Given that, I believe it is crucial to scrutinize both single and combined stress in sensitive and tolerant potato varieties for the authenticity of the research. Considering the significant value of the findings in this study, particularly with regard to combined stress, I believe it should be accepted without requiring any further scientific revisions.

Corresponding author: Native speaker's help was sought for grammatical corrections and final language proofreading. Several sentences have been rephrased to improve language quality and clarity.

Responses To Reviewer 2

Query: The sentence beginning with "It is worth noting that plants exhibit ...." Is difficult to understand. Please make it clear. (Lines 71-73).

Corresponding Author: The sentence has been revised to make it more understandable. (Lines 71-73)

Query: Please check and add recent publications to strengthen the hypothesis for the interconnection between light and drought mechanisms. (Lines 74-76)

Corresponding Author: The premise that a putative crosstalk between the light signaling mechanism and drought stress response in our study has been reinforced by the addition of two recent articles (Mukherjee et al. 2023; Wang et al. 2023) in the draft. The respective citations have been added to the reference section at the end of the manuscript and highlighted (Lines 74-76).

Query: Correct "moso bamboo" as "Moso bamboo". (Line 83)

Corresponding Author: Relevant changes have been made in the specified section and highlighted. (Line 83)

Query: Correct "salinity toleranca" as "salinity tolerance". (Line 96)

Corresponding Author: The relevant word has been corrected, as requested and highlighted. (Line 96).

Query: Change "internal control" to "housekeeping gene". (Line 217)

Corresponding Author: The relevant corrections have been made in the manuscript. (Line 217).

Query: Add the sequence information of the housekeeping gene to Table S1 in the supplementary file. (Line 217)

Corresponding author: The sequence information and other related information of the housekeeping gene for RT-qPCR have been added to Table S1 in the Supplementary File and highlighted. (Table S1 Supplementary File).

Query: Correct "StgGATA" with "StGATA". (Line 235)

Corresponding author: The relevant correction has been made in the manuscript. (Line 235).

Query: Can you write the number of StGATAs present in chromosome I in a sentence? (Lines 266-267)

Corresponding author: The number of StGATAs present on chromosome I has been already mentioned in the text. (Lines 266-267).

Query: Please make the sentence clear starting with "Unfortunately, grouping according to gene structure did...". What do you try to explain? (Lines 244-245)

Corresponding author: The sentence has been revised to make it more understandable and highlighted. (Lines 244-245).

Query: Can you please very briefly mention the details for the function of AtGATA03 and AtGATA29 in the discussion part? (Lines 410-416)

Corresponding author: Extra information has been added to explain the functions of AtGATA03 and AtGATA29, and highlighted. (Lines 410-416 in discussion part)

Query: Please add numeric data for the phenotypic observations. (Line 341; Lines 346-347)

Corresponding author: Numeric data have been added to respective places. (Line 341; Lines 346-347)

Query: You mentioned that "Sante was relatively tolerant to light". Some information is missing here. Which particular light? (Line 342)

Corresponding author: The relevant correction has been made in the manuscript. (Line 342).

Query: Correct "25-folds" with "25-fold". (Line 352).

Corresponding author: The relevant correction has been made in the manuscript. (Line 352).

Query: The sentence that starts with "Although our sub-groups did not exhibit conserved gene structure patterns" is not clear. Please make it clear. (Lines 420-422)

Corresponding author: The sentence has been revised to make it more understandable and highlighted. (Lines 420-422).

Query: Is there a typo in the sentence that starts with "Unlike Group IIB, Group II comparably...."? I think there is a mistake here. (Line 492)

Corresponding author: There is no typo; however, the information is missing. Groups IIB and IIC had the largest number of interacting proteins in Group II. This is emphasized in the respective lines. (Line 492).

Query: Please give units to shoot and root lengths in Figure S6 in the supplementary file.

Corresponding author: The relevant addition has been made in Figure S6 in the Supplementary File and is highlighted.

Extra revisions

Lines 16-22 (in Abstract). The abstract has been revised after further *in silico* PPI and qPCR analyses.

Lines 147-148 (in Materials and Methods). The interpretation of *cis*-element data was improved with the help of TBtools, and the new figure was given in Figure 6 (the figure caption was highlighted).

Lines 172-181 (in Materials and Methods). The methodology for PPI and co-expression network analyses is also provided.

Lines 213-215 (in Materials and Methods). The UniProt and NCBI accession numbers of interacting proteins are provided in the text.

Line 333 (in Results). “Selected StGATAs” is added to the title in order to add the results of further experiment.

Lines 367-398 (in Results). The results of qPCR analyis are provided for interacting proteins.

Lines 546-587 (in Discussion). The results of qPCR analyis were discussed.

The bar colors were changed in **Figure 8** in order to make the standard error lines visible and clear. The former version had black and white bars. (Figure file)

Figure 9 was generated to show the expression of genes interacting with StGATAs. (Figure file)

Primer list in **Table S1** was revised after new experiments for M1AZB3, M0ZT32, M0ZL05, M1CSN7, and M1AHQ7. (Supplementary file)

Protein-protein interaction analysis of selected StGATAs were provided in **Figure S3**. (Supplementary file)

The table for the annotation of selected StGATA interacting proteins was given in Supplementary file in **Table S2**.
